# “CinNapht” dyes: a new cinnoline/naphthalimide fused hybrid fluorophore. Synthesis, photo-physical study and use for bio-imaging†

**DOI:** 10.1039/d1ra05110e

**Published:** 2021-09-08

**Authors:** Minh-Duc Hoang, Jean-Baptiste Bodin, Farah Savina, Vincent Steinmetz, Jérôme Bignon, Philippe Durand, Gilles Clavier, Rachel Méallet-Renault, Arnaud Chevalier

**Affiliations:** Université Paris-Saclay, CNRS, Institut de Chimie des Substances Naturelles UPR 2301 91198 Gif-sur-Yvette France arnaud.chevalier@cnrs.fr; Université Paris-Saclay, CNRS, Institut des Sciences Moléculaires d’Orsay Orsay 91405 France; Université Paris-Saclay, ENS Paris-Saclay, CNRS, PPSM 91190 Gif-sur-Yvette France

## Abstract

Six-membered-diaza ring of cinnoline has been fused on naphthalimide dye to give a donor–acceptor system called CinNapht. This red shifted fluorophore, that can be synthesised in gram scale, exhibits a large Stoke shift and a fluorescence quantum yield up to 0.33. It is also characterized by a strong solvatochromic effect from green to red emission as well and can be used for bio-imaging.

## Abstract 2

Six-membered-diaza ring of cinnoline has been fused on naphthalimide dye to give a donor–acceptor system called CinNapht.

## Graphical abstract

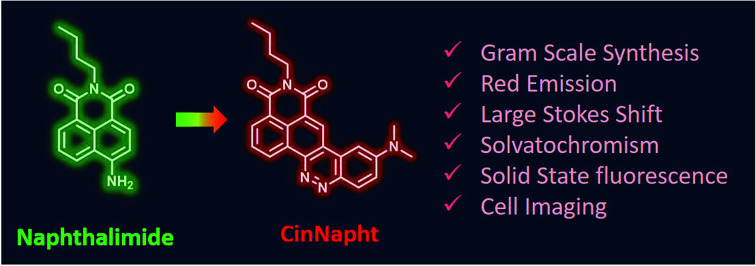

With the increasing interest of optical molecular imaging in medicine, fluorescence microscopy has seen constant development contributing to the emergence of new technologies and probes without discontinuity for nearly 40 years. Fluorogenic probes are now considered as critical tools for the study of biological environments.^1^ These sensors unmask brilliant fluorescence upon modification of their structure or of their environment, induced by multiple kinds of biological stimuli.^2^ Therefore, there is definite interest in creating a new chemical scaffold exhibiting fluorescent behavior that could later be used for the design of chemical fluorogenic probes. One of the most important and actual challenges is the synthesis of large Stokes shift red emitting fluorophores.^3^ Among the different families of fluorophores commonly used, the 1,8-naphthalimides such as 4-amino-1,8-naphthalimide (ANI) exhibit a green emission combining good fluorescence quantum yield and large Stokes shift (∼4000 cm^−1^) while possessing a particularly high chemical stability.^4^ 1,8-Naphthalimide-based sensors in solution or as part of a material have been used for the detection of multiple analytes such as anions, metals or enzymes, highlighting their interest for fluorescence imaging.^5^ The easy functionalization of their imide nitrogen atom enables fine tuning of the fluorophore properties such as solubility or organelle targeting.^6^ However, their uses in imaging experiments are limited to the green region of the spectrum. To overcome this drawback, some groups have focused on the synthesis of π-extended naphthalimides fluorophores such as styrylnaphthalimides.^7^ The latter were found to be very promising red to NIR emitting dyes and were used for designing fluorogenic probes.^8^

Other examples including a fused pyranone, furan or carbazole rings^9^ have also been reported but with less interesting photophysical properties. Nonetheless, this strategy seems promising for there are many examples of red shifted fused hybrids fluorohores.^10^ We describe in this paper the synthesis of a fused ring cinnoline/naphthalimide hybrid here called CinNapht dye. Cinnolines are aromatic heterocycles incorporating an azobenzene moiety that usually do not emit fluorescence, mainly because of nonradiative deactivation through photoisomerization of the azo bond.^11^ These molecules can nevertheless be turned into fluorescent structures by constraining the conformation of the N=N double bond like it is in cinnoline scaffold.^12^ A significant number of fluorophores have recently been described incorporating this scaffold. Nevertheless many of these examples have required quaternization of the nitrogen atom to generate a push–pull electron effect, and induce an intramolecular charge transfer (ICT) necessary for fluorescence emission.^13^ More recently, fluorogenic probes in which a cinnoline is an integral part of a push–pull backbone have been developed. In these cases the azo bond is formed *in situ* following nitric oxide mediated nitrosation of an aniline precursor according to the “covalent assembly” principle, firstly proposed by Anslyn in 2010 ^14^ and then exemplified for multiple times.^15^ To the best of our knowledge, only one of these probes is formed with a molecular skeleton incorporating a naphthalimide like dye.^15*a*,*b*^ However, no description of its photophysical properties is given. Moreover, it was not isolated and such strategy is limited to the NO detection. We will show in this article that this type of molecule can nevertheless possess original photophysical properties that deserve to be highlighted. We first proceeded with the synthesis of these fused naphthalimide cinnoline hybrid (Fig. 1).

**Fig. 1:**
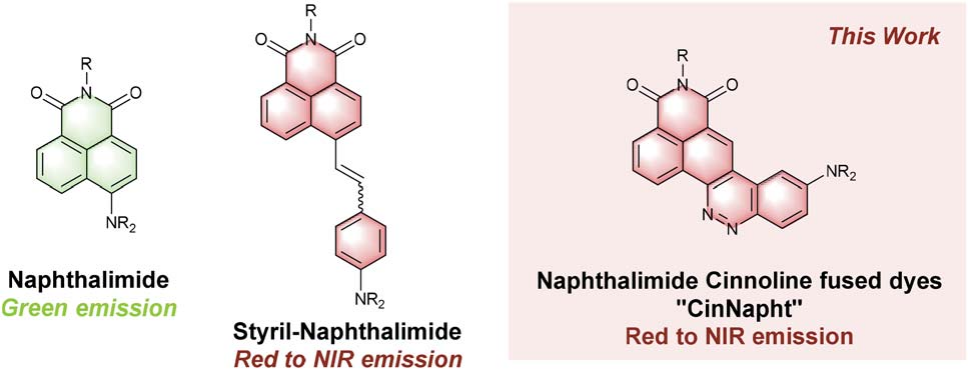
Structure of naphthamidide dyes and extended analogues.

4-Amino-1,8-naphthalic anhydride 1 (Scheme 1) was first synthesized from commercial 4-bromo analog by a two steps process involving an aromatic nucleophilic substitution with sodium azide followed by reduction under Staudinger conditions (*cf.* ESI†). This aniline was then brominated in *ortho* position using *N*-bromosuccinimide in hexafluoroisopropanol (HFIP)^16^ to provide the intermediate 2 with 92% yield, and the naphthalimide 3 was formed by reaction with *n*-butylamine in the yield of 92%. It should be mentioned that no purification was needed for both of these two steps which greatly facilitates the synthetic process. The biaryl 4 was then obtained by Suzuki coupling of 3 with 3-(dimethylamino)phenylboronic acid in the yield of 82%. Under the diazotization conditions, NaNO_2_/dilute HCl, 4 was converted in a mixture of two regioisomers resulting either from an azo coupling reaction in *para* position (5a) or in *ortho* position (5b) in a 84 : 16 ratio (determined by HPLC analysis of the crude mixture, Fig. S1†). The predominant CinNapht 5a was obtained with only 58% yield after purification. The minor compound 5b was obtained in the yield of 16% and exhibited a very weak fluorescence emission that we decided not investigate further. By contrast, CinNapht 5a was found to be much more emissive. In order to optimize its synthesis, azo coupling reaction of 4 was attempted with NOBF_4_ as diazotization reagent in CH_3_CN. It turned out that this method enabled the almost exclusive formation of the compound 5a. HPLC analysis showed a 96 : 4 ratio in favor of CinNapht 5a (Fig. S2†) which could be isolated after purification in the yield of 73% with a high purity of 98.5% (Fig. S3†). The complete synthetic pathway was also reproduced with success in large scale in order to demonstrate its viability for gram scale synthesis of the CinNapht dyes.

**Scheme 1:**
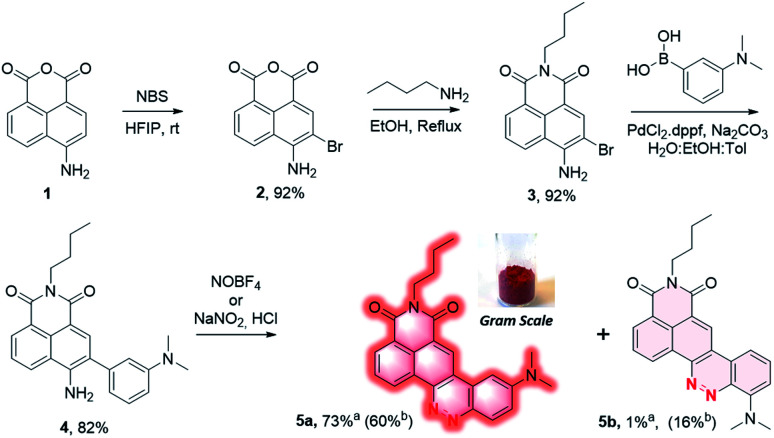
Synthesis of CinNapht 5a end 5b. ^a^Isolated yield using NOBF_4_. ^b^Isolated yield using NaNO_2_/HCl.

We then performed a complete analysis of the photophysical properties of CinNapht 5a in different solvents. Absorption, excitation and emission spectra were recorded, molar extinction coefficient (*ε*) (Fig. S4†), fluorescence quantum yields and fluorescence life times (Fig. S5†) were measured (*cf.*Table 1). All spectra and data are presented in ESI (Fig. S6 to S15†). The first observation is a significant red shift in the emission wavelength relative to the initial naphthalimide fluorophores. Thus an 89 nm bathochromic shift of the *λ*_max_ Em was observed between ANI (*λ*_max_ Em = 502 nm) and CinNapht 5a (*λ*_max_ Em = 591 nm) in DCM (*cf.* Fig. S16†). Fluorescence quantum yields (QY) were found to be mostly modest in all tested solvents with nevertheless values up to 0.33 in DCM and a 3571 cm^−1^ Stokes shift which is quite satisfying.^19^ The QY heterogeneity might be the reflect of some kind of aggregation phenomenon for which a better solubility observed in CHCl_3_ or DCM leads also to a better quantum yield as well as a better life time decay. This could also explain the difference of fluorescence quantum yield observed in RPMI medium supplemented (entry 10, Table 1) or not (entry 9, Table 1) with 10% of FBS. Another important characteristic of this fluorophore is the possibility of exciting it at higher energy on the S0 → S2 transition band (Fig. S17†). This allows to significantly enlarge the difference between the absorption and emission maxima, which then reaches values exceeding 10 000 cm^−1^. Finally, we also observed a solid-state fluorescence of 5a with an orange-red emission centered at 581 nm (Fig. S18†). As the push–pull structure of the compound suggested an ICT type fluorescence, a solvatochromic study was carried out. By lighting at 365 nm solutions of CinNapht 5a in different solvents, a color panel ranging from green to red-pink was obtained, likely correlated with the solvent polarity (Fig. 2). This was confirmed by the linear relationship between the *λ*_max_ Em of CinNapht 5a and the polarity coefficient *E*_T_(30) of the solvents with an exception for DMSO, (Fig. S19†).^17^ This partial lack of adequacy could be explained by the fact that the Dimroth and Reichardt method does not integrate any basicity parameter, which is particularly high in the case of DMSO.

**Table 1:** Photophysical properties of CinNapht 5a

Entry	Solvent	*E*_T_(30)^a^	*λ*_max_ abs^b^ (nm)	*ε*_max_ (M^−1^ cm^−1^)	*λ*_max_ Em (nm)	Stokes shift (cm^−1^)	QY^c^	Life time (ns)
1	Hexane	31.0	469	12 200	520	2091	0.01	0.37
2	Toluene	33.9	480	12 000	550	2652	0.09	0.88
3	Dioxane	36.0	477	21 000	572	3482	0.16	1.89^d^
4	CHCl_3_	39.1	489	15 700	566	2782	0.25	2.23
5	DCM	40.7	488	16 400	591	3571	0.33	3.67
6	DMSO	45.1	496	15 500	682	5499	0.06	1.26^d^
7	EtOH	51.9	493	15 200	667	5291	0.05	0.84
8	MeOH	55.4	492	15 100	681	5640	0.02	0.47^d^
9	RPMI^e^	n.a.	496	n.d.^f^	675	5346	<0.01	n.d.
10	RPMI^e^ + 10% FBS	n.a.	493	n.d.^f^	651	4923	0.02^g^	n.d.
11	In cell	n.a.	475	n.a.	590	4104	n.d.	n.d.
12	Solid	n.a.	491	n.a.	581	3446	n.d.	n.d.

a Polarity coefficient of solvents based on literature.^17^

b Values corresponding to S_0_–S_1_ transition but strong S_0_–S_2_ transition is also observed (see ESI Fig. S6 to S13).

c Relative QY determined at 25 °C using [Ru(bpy)_3_]Cl_2_ (QY = 0.04 in air saturated H_2_O).^18^

d Fitted by a biexponential function, the value indicated is an average lifetime. (For more details see ESI Fig. S5 and experimental section). In dioxane the two lifetime were 1.66 ns and 3.31 ns. In DMSO the lifetime were 1.13 ns and 2.21 ns. In MeOH the lifetime were 2.68 ns and 0.42 ns.

e Solubility in aqueous medium such as RPMI culture medium (Roswell Park Memorial Institute medium), without phenol red, was found to be quite poor and required 5% of DMSO.

f A small part of precipitation was observed that could not enable an accurate determination of *ε*.

g Values observed by adding 10% of Foetal Bovine Serum (FBS) in RPMI medium.

**Fig. 2:**
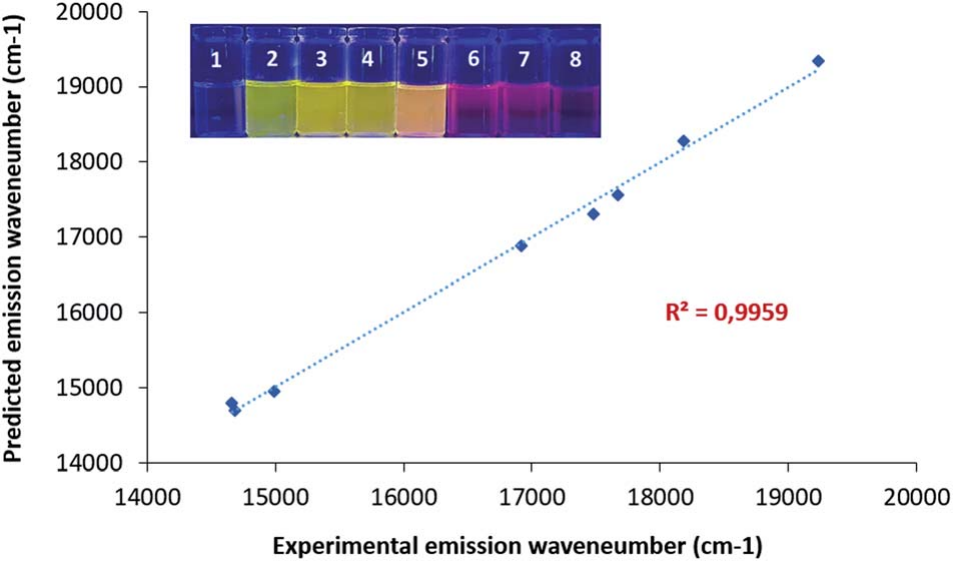
Solvatochromism study: visual representation of CinNapht solution under 365 nm light. (1) Hexane, (2) toluene, (3) CHCl_3_, (4) dioxane, (5) DCM, (6) EtOH, (7) MeOH, (8) DMSO) and plot of experimental and predicted emission wavenumber (Catalan method).

In order to confirm this, the solvent effect was also analyzed with the methodology developed by Catalán^20^ that relies on the description of the solute–solvent interactions with four independent parameters: polarizability (SP), dipolarity (SdP), acidity (SA) and basicity (SB).

The solvent-dependent maximum emission wavenumber (*ν̄*) is formulated in eqn (1) as:

1 *ν̄* = *ν̄*_0_ + *a* × SP + *b* × SdP + *c* × SA + *d* × SB

where *ν̄*_0_ is the value of the property in the gas phase, and the coefficients *a*–*d* are the regression coefficients describing the sensitivity of the wavenumber to the different solute–solvent interactions. The fit of the experimental fluorescence wavenumbers in the various solvent gives:

2 *ν̄* = 19950(±54) − 725(±40)SP − 2503(±24)SdP − 1506(±3)SA − 3012(±67)SB

showing that the fluorescence of CinNapht is mainly dependent on dipolarity, acidity and basicity parameters (see complete values Fig. S20†). Eqn (2) together with the linear correlation between calculated *ν̄* and experimental *ν̄* (graph, Fig. 2) confirmed the solvatochromic behavior of the CinNapht 5a which is in accordance with its push–pull structure suggesting that ICT is responsible for the emission. To complete the experimental study and rationalize the photophysical properties, (TD)DFT calculations were carried out (Fig. 3). The geometry of the CinNapht dye is flat and the first transition (S0 → S1) happens from the HOMO to the LUMO. The HOMO is centred on the amino-cinnoline part while the LUMO is more localized on the naphthalimide part of the dye. This result together with the representation of the density difference confirm the intramolecular charge transfer (ICT) nature of the first excited state (Fig. S21†). The possibility of a twisted induced charge transfer (TICT) excited state was also evaluated but the energy level of that state is higher than the ICT one (*E*(S1-TICT) > *E*(S1-ICT)) even when solvent effect was included.

The solvatochromic effect observed in the fluorescence spectra is thus coming from solvent effects and not from the transition from a locally excited state to a TICT one.

Finally, we have validated the potential of these new fluorophores in cell imaging experiments. CinNapht 5a was incubated at 5 μM with living A549 cells for 2 h.

The images presented Fig. 4 demonstrate the viability of our fluorophores for cell imaging. No pretreatment was necessary to enable cell penetration thereby strengthening the potential of CinNapht for fluorescence microscopy. Emission and excitation spectra were recorded in cell during microscopy experiments (Fig. S22†) and revealed that CinNapht 5a exhibits a Stokes shift large enough to enable an excitation at 475 nm (maximum of excitation measured in cell) while recording fluorescence across the full range of emitting fluorescence from 500 to 700 nm centered at 591 nm. We emphasis that at the concentration used for these cell imaging experiments, fluorophore 5a showed no toxicity (see Fig. S23†). Complete cytotoxicity study was carried out and did not reveal any significant toxicity even at high concentration (50 μM) (Fig S23†).

**Fig. 3:**
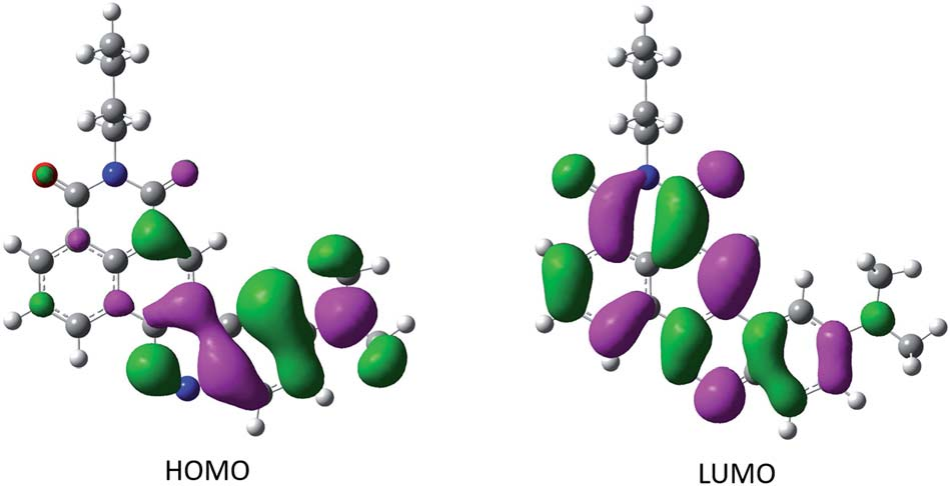
Isocontour plot of the HO and LU molecular orbitals (isovalue 0.004).

**Fig. 4:**
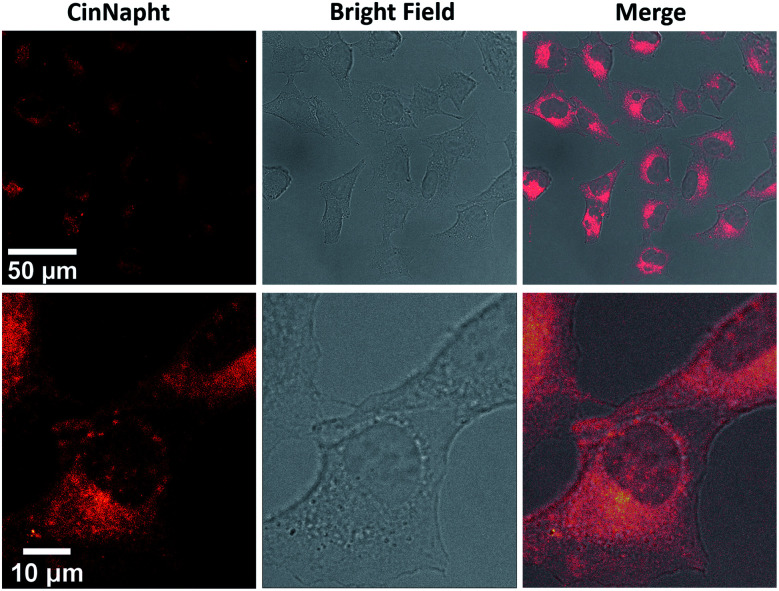
Confocal microscopic images of A549 lung cancer cells treated with CinNapht 5a at 5 μM for 2 h at 37 °C using a 40× oil immersion objective. (Exc: 475 nm Em: 500–700 nm).

We did not notice any significant photobleaching of the dye during these microscopy experiments. However, we cannot claim a strong photochemical stability of our fluorophore at this stage of the study. To conclude, we describe here a new fluorophore based on fused naphthalimide cinnoline hybrid structure so called “CinNapht”. This fluorophore shows promising fluorescence properties combining a red emission and a large stokes shift. Calculations have confirmed an ICT-like behavior characteristic of a push–pull structure that is clearly identified in the skeleton of the CinNaphth. This characteristic is reflected in the photophysical properties by a strong solvatochromism effect. This non-toxic molecule can be used for *in cellulo* imaging experiments. We believe that an optimization of the structure of CinNaphth, for example by modifying the *N*-dimethyl moiety, should allow a significant improvement of the photophysical properties of these fluorophores, thus making them promising tools for cell imaging.

## Conflicts of interest

There are no conflicts to declare.

## Supplementary Material

RA-011-D1RA05110E-s001
