# Supplementary material for: “CinNapht” dyes: a new cinnoline/naphthalimide fused hybrid fluorophore. Synthesis, photo-physical study and use for bio-imaging
Source: RSC Adv. 2021 Sep 8;11(48):30088–92. doi: 10.1039/d1ra05110e (PMC9041350; doi:10.1039/d1ra05110e)
Supplement: RA-011-D1RA05110E-s001 [file RA-011-D1RA05110E-s001.pdf]

Electronic Supplementary Material (ESI) for RSC Advances.
This journal is © The Royal Society of Chemistry 2021

"CinNapht" dyes a new Cinnoline/Naphthalimide fused hybrid fluorophore: Synthesis, Photo-physical study and use for bio-imaging

Minh-Duc Hoang,[a‡] Jean-Baptiste Bodin,[b‡] Farah Savina,[b] Vincent Steinmetz,[a] Jérôme Bignon,[a] Philippe Durand,[a] Gilles Clavier,[c] Rachel Méallet-Renault[b] and Arnaud Chevalier*[a]

[a]*Institut de Chimie des Substances Naturelles, CNRS UPR 2301, Université Paris-Saclay, 1, Avenue de la Terrasse, 91198 Gif-sur-Yvette, France*
[b]*Université Paris-Saclay, CNRS, Institut des Sciences Moléculaires d'Orsay, Orsay, 91405, France*
[c]*PPSM, CNRS, ENS Paris-Saclay, 91190, Gif-sur-Yvette, France*

E-mail: arnaud.chevalier@cnrs.fr

‡ These authors contributed equally to this work

Supporting Information

I. Additional figures 4

Fig S1: HPLC Analysis (System A) of crude mixture using $NOBF_4$ as nitrosylation system 4

Fig S2 HPLC Analysis (System A) of crude mixture using $NaNO_2$/HCl as nitrosylation system 5

Fig S3: Quality control report of CinNapht **5a** using RP-UPLC-MS (System B) 6

Fig S4: Epsilon Calculation in different solvents 6

Fig S5: Time Decay Analysis 7

Fig S6: Spectral properties of CinNapht **5a** in n-Hexane: 8

Fig S7: Spectral properties of CinNapht **5a** in Toluene: 9

Fig S8: Spectral properties of CinNapht **5a** in $CHCl_3$: 9

Fig S9: Spectral properties of CinNapht **5a** in Dioxane: 10

Fig S10: Spectral properties of CinNapht **5a** in DCM: 10

Fig S11: Spectral properties of CinNapht **5a** in Ethanol: 11

Fig S12: Spectral properties of CinNapht **5a** in Methanol: 11

Fig S13: Spectral properties of CinNapht **5a** in DMSO: 12

Fig S14: Superposition of emission spectre of Naphthalimide and CinNapht **5a** in DCM 13

Fig S15: Emission spectra obtained with excitations at 343 nm and 488 nm in chloroform and Excitation spectrum for an emission centered at 585 nm 14

Fig S16: Solid State fluorescent properties of CinNapht **5a** 14

Fig S17: Correlation between Maximum emission wavelength and corresponding solvent polarity coefficient $E_T(30)$ 15

Fig S18: Table of complete values relative to Catalan method calculations[1].. 15

Fig S19: TD(DFT calculation) 16

Fig S20: Excitation and Emission Spectra in living cell.. 16

Fig S21: Cytotoxicity Study 17

II. Abbreviations 18

III. Experimental section 18

General 18

Instrument and methods.. 18

Spectroscopy Studies 19

Luminescence lifetime.. 19

High-performance liquid chromatography separations.. 20

Quantum chemical calculations 20

Cell culture.. 20

Fluorescence microscopy 21

Cytotoxicity Study.. 21

Synthesis protocols.. 21

IV. NMR and MS Spectra.. 25

^{1}H NMR of compound **1**.. 25

^{13}C NMR of compound **1**.. 25

HRMS (ESI+) of compound **1** 26

^{1}H NMR of compound **2**.. 26

^{13}C NMR of compound **2**.. 27

HRMS (ESI+) of compound **2** 27

^{1}H NMR of compound **3**.. 28

^{13}C NMR of compound **3**.. 28

HRMS (ESI+) of compound **3** 29

^{1}H NMR of compound **4**.. 29

^{13}C NMR of compound **4**.. 30

HRMS (ESI+) of compound **4** 30

^{1}H NMR of compound **5a**.. 31

^{13}C NMR of compound **5a**.. 31

HRMS (ESI+) of compound **5a** 32
^{1}H NMR of compound **5b** 32
^{13}C NMR of compound **5b** 33
HRMS (ESI+) of compound **5b** 33
Optimized geometry of **5a** 34
V. References 35

I. Additional figures

Fig S1: HPLC Analysis (System A) of crude mixture using $NOBF_4$ as nitrosylation system

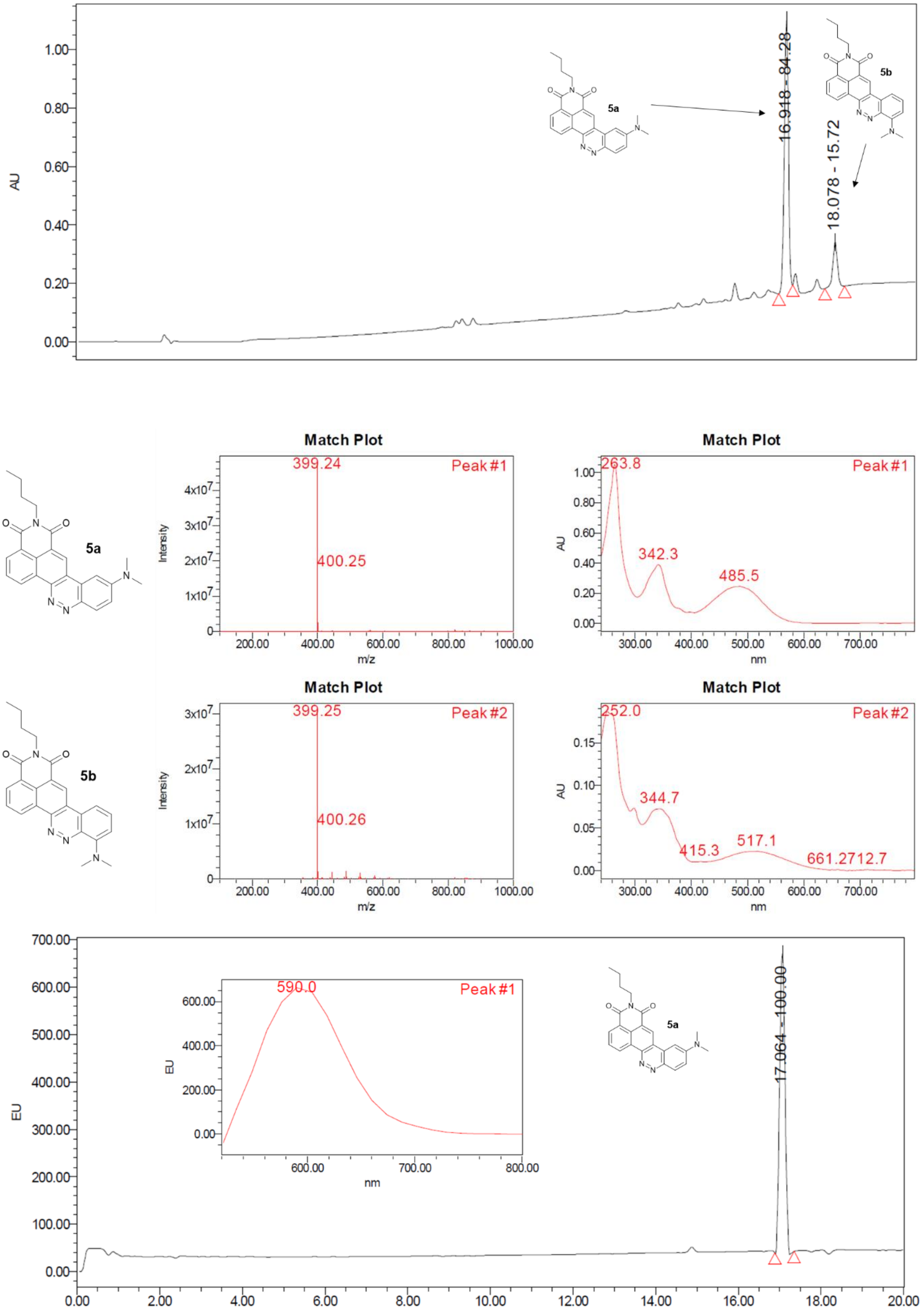

Fig S2 HPLC Analysis (System A) of crude mixture using $NaNO_2$/HCl as nitrosylation system

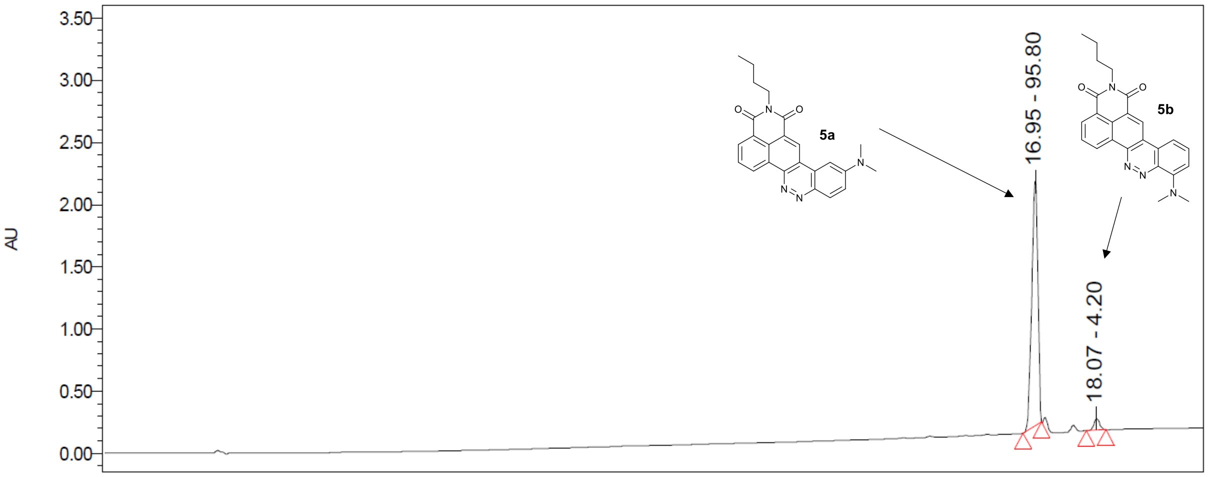

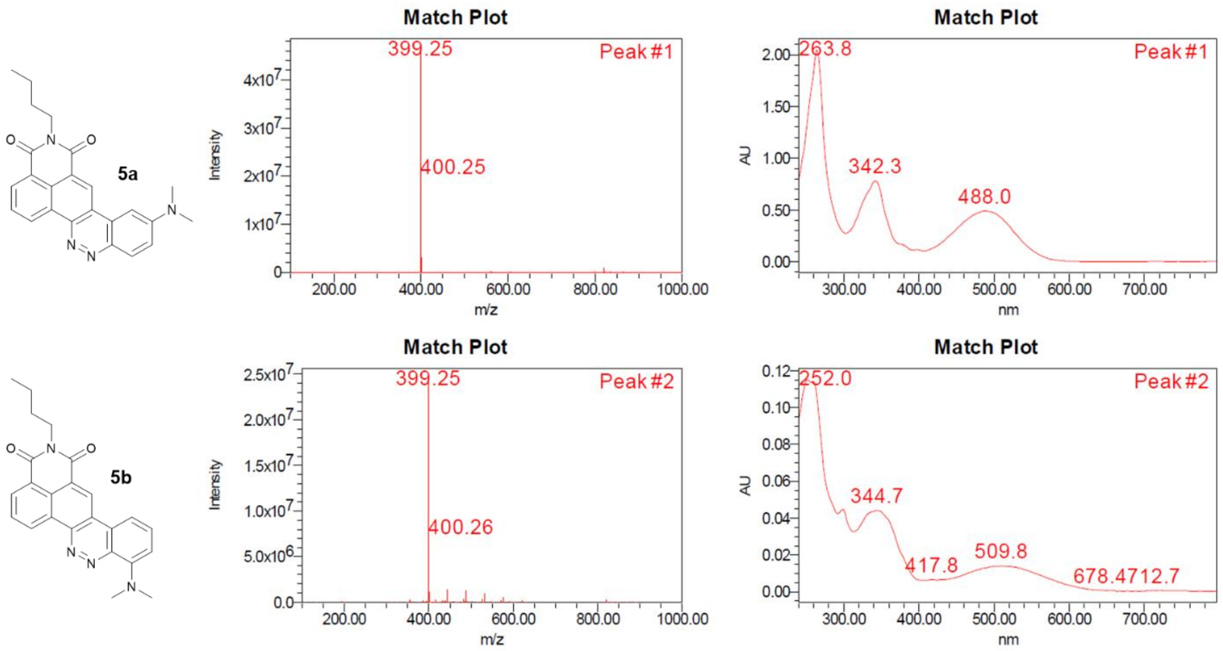

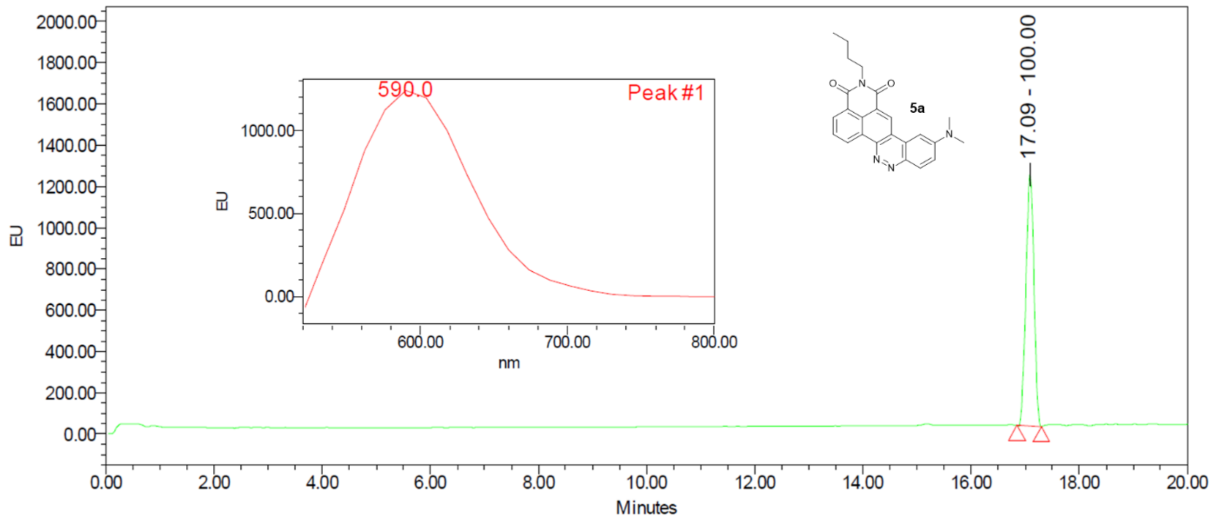

Fig S3: Quality control report of CinNapht 5a using RP-UPLC-MS (System B)

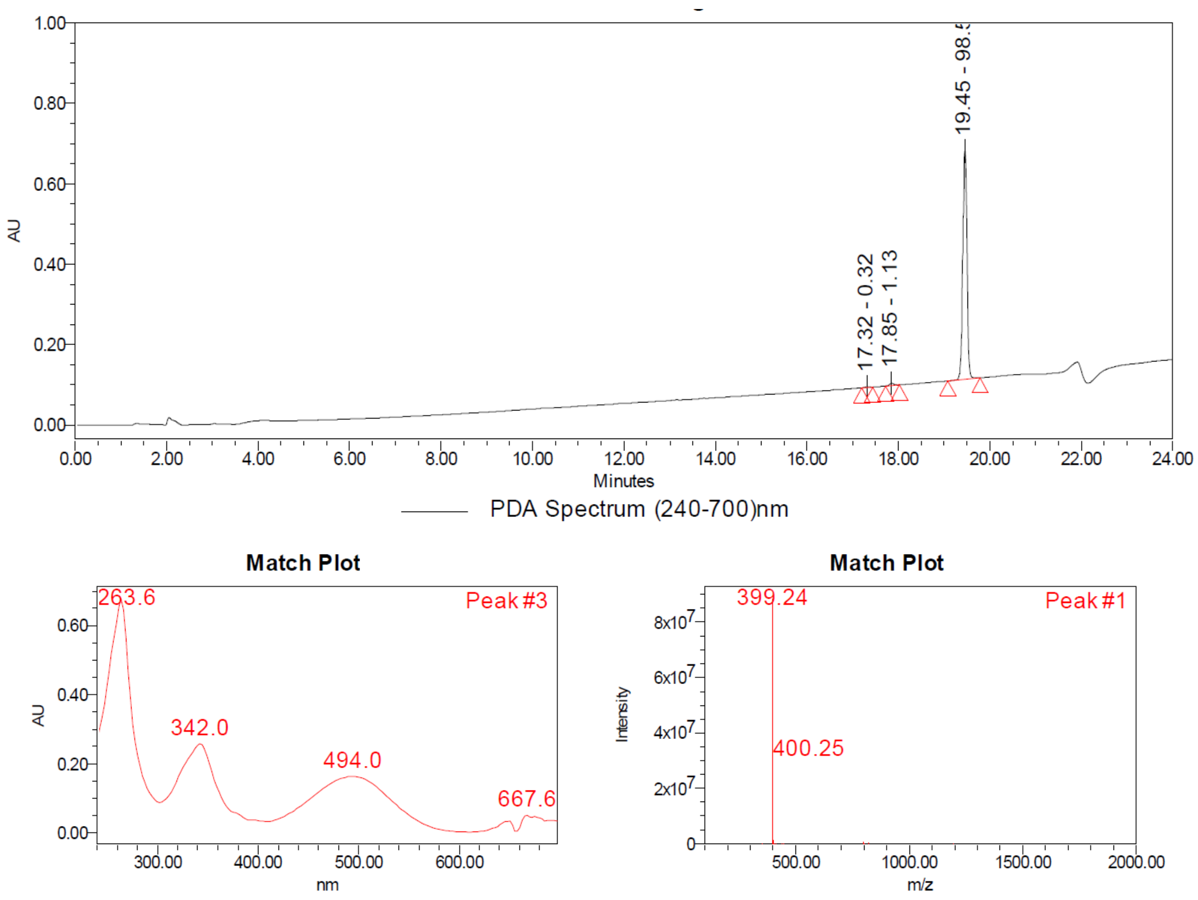

Fig S4: Epsilon Calculation in different solvents

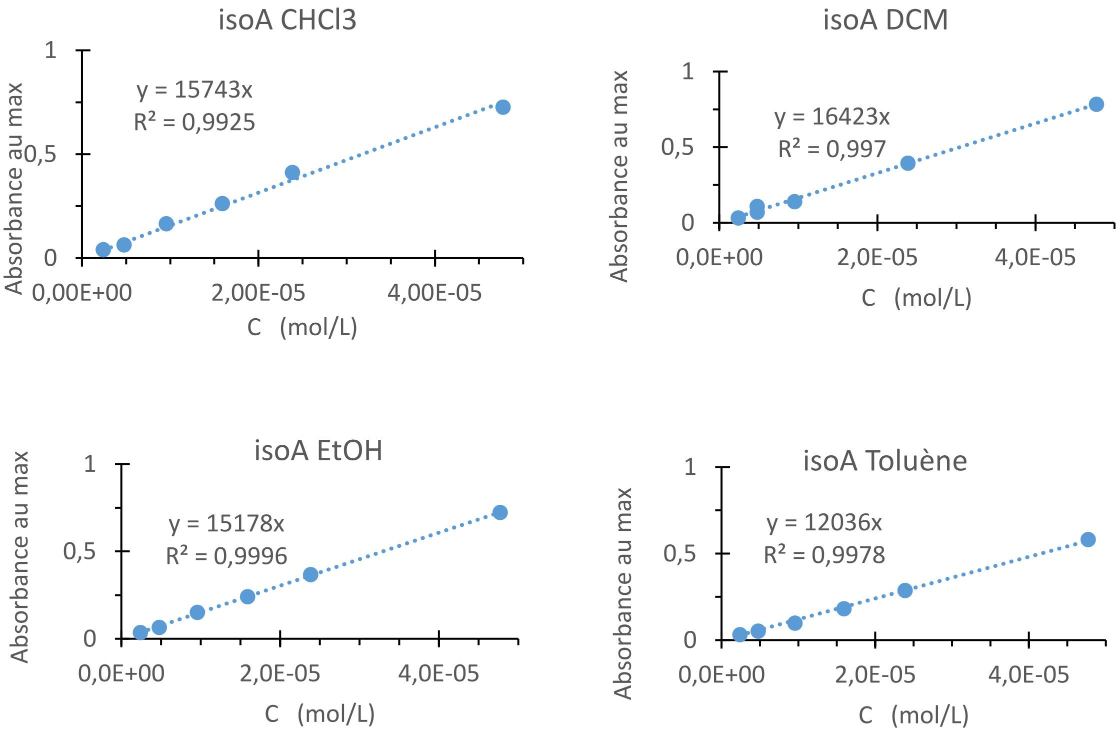

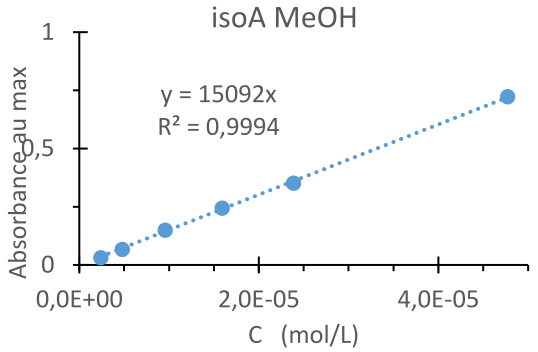

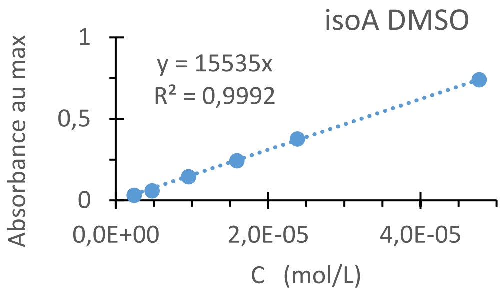

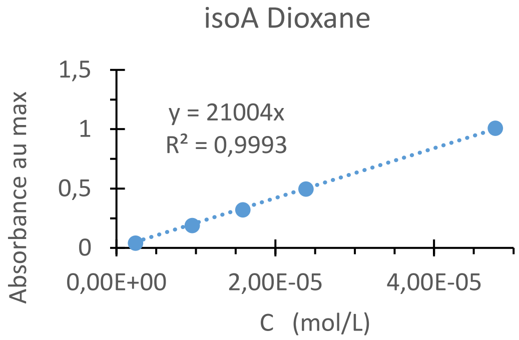

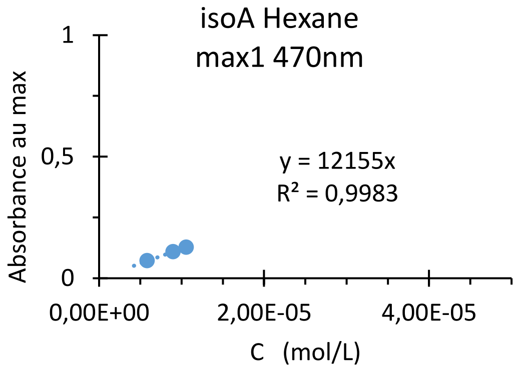

Fig S5: Time Decay Analysis

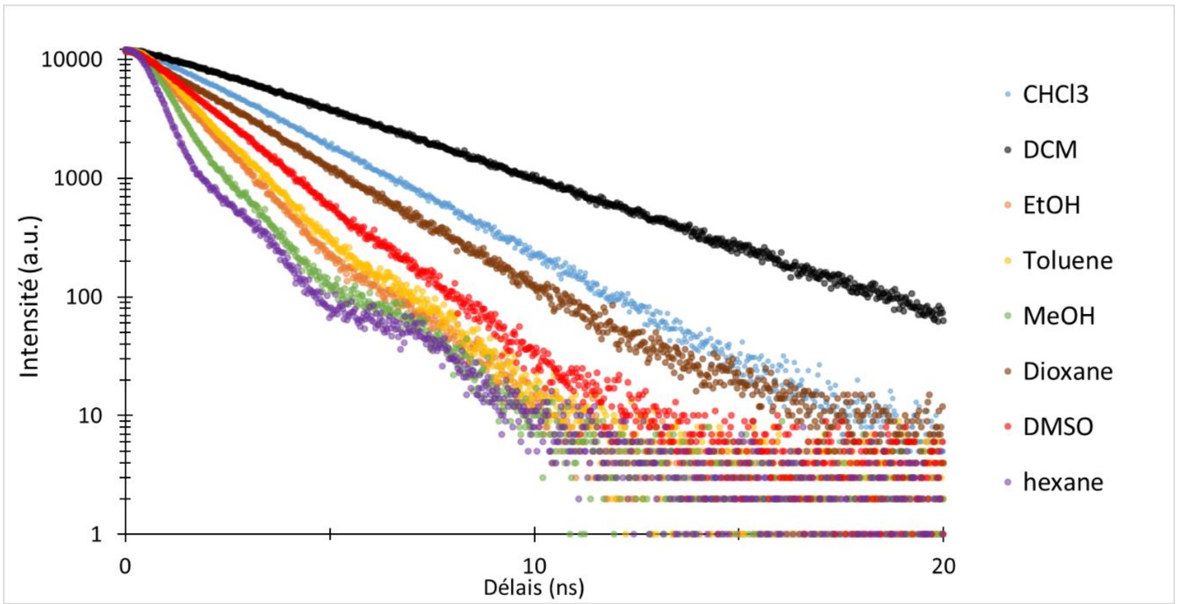

DMSO	τ (s)	relative amplitude	Normalized pre-exp	Average τ (s)
Life time 1	1,13E-09	88,1 %	93,5 %	1,26E-09
Life time 2	2,2E-09	11,9 %	6,5 %	
dioxane	values	relative amplitude	Normalized pre-exp	Average τ
Life time 1	1,659E-09	86,1 %	92,5 %	1,89E-09
Life time 2	3,3E-09	13,9 %	7,5 %	
MeOH	values	relative amplitude	Normalized pre-exp	Average τ
Life time 1	4,204E-10	97,9 %	99,7 %	4,67E-10
Life time 2	2,7E-09	2,1 %	0,3 %	

Fig S6: Spectral properties of CinNapht 5a in n-Hexane:

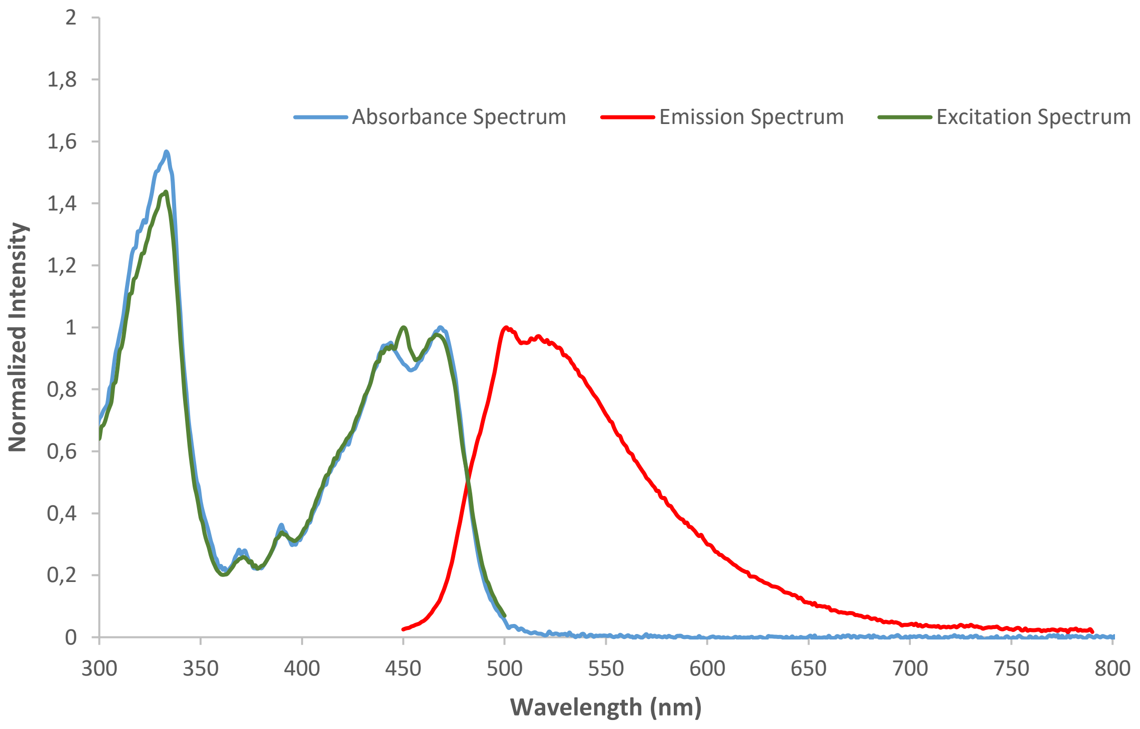

Fig S7: Spectral properties of CinNapht 5a in Toluene:

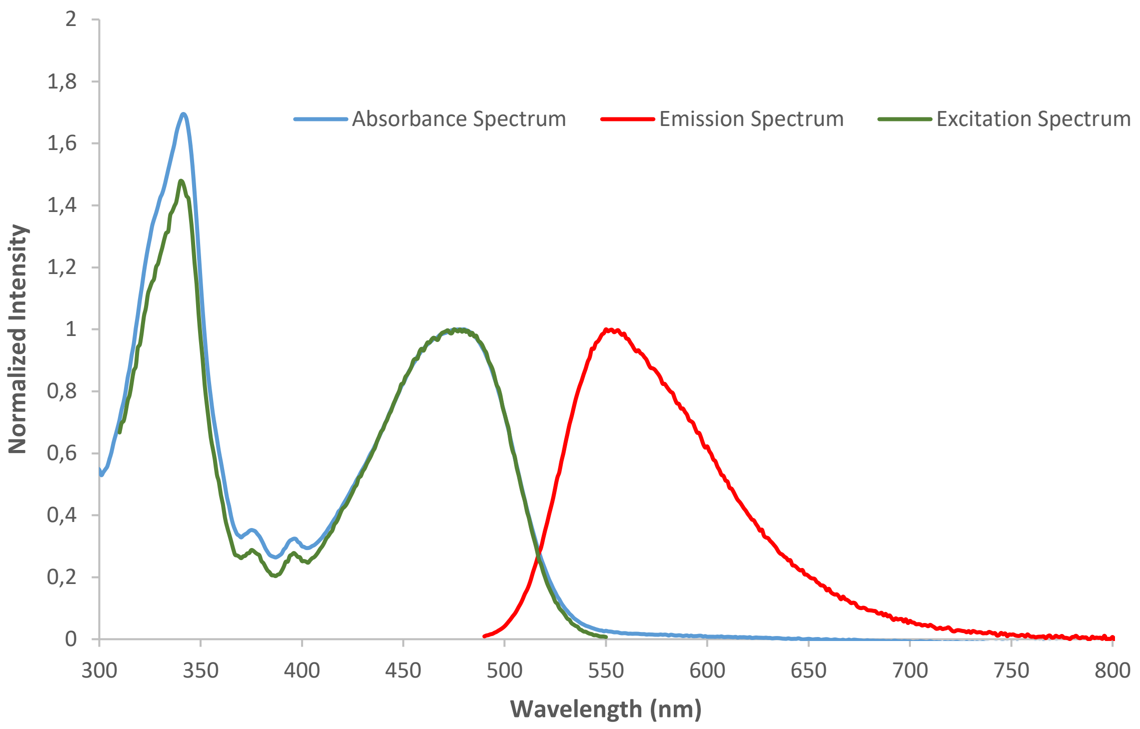

Fig S8: Spectral properties of CinNapht 5a in $CHCl_3$:

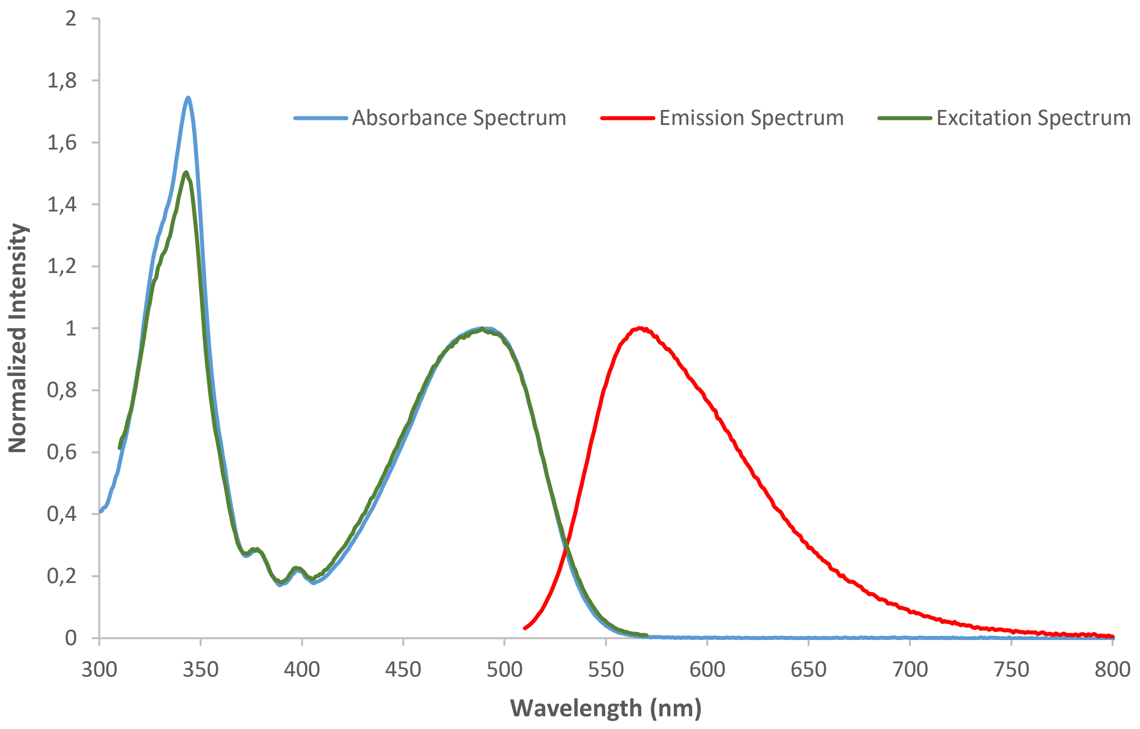

Fig S9: Spectral properties of CinNapht 5a in Dioxane:

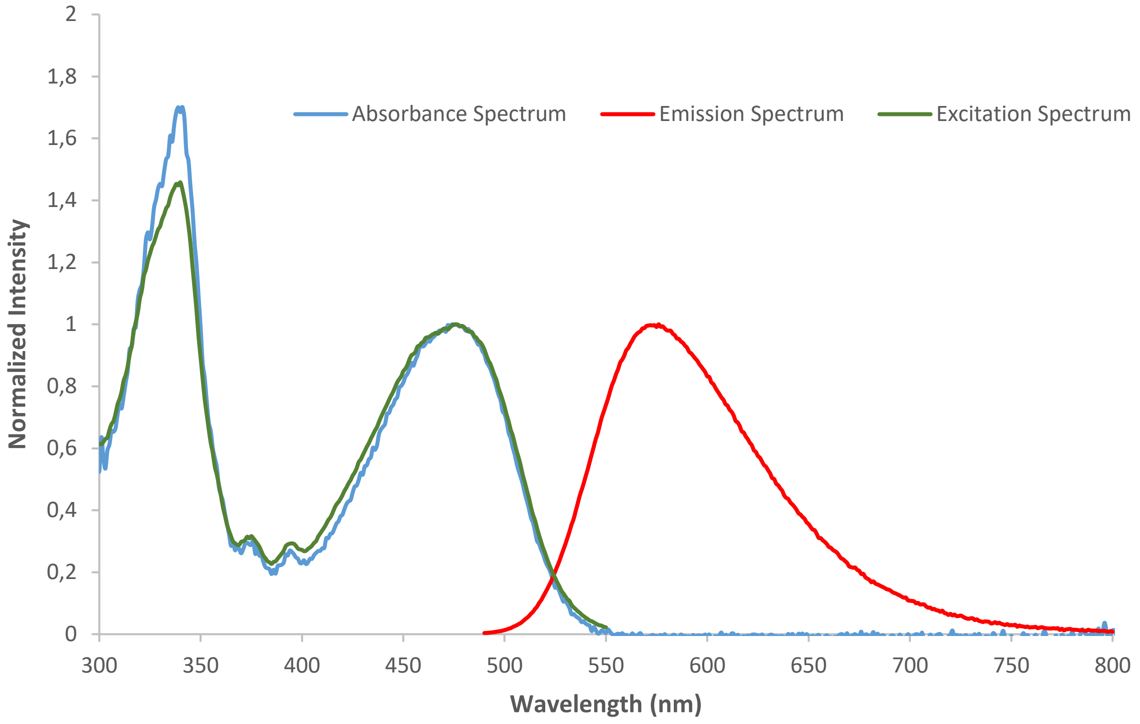

Fig S10: Spectral properties of CinNapht 5a in DCM:

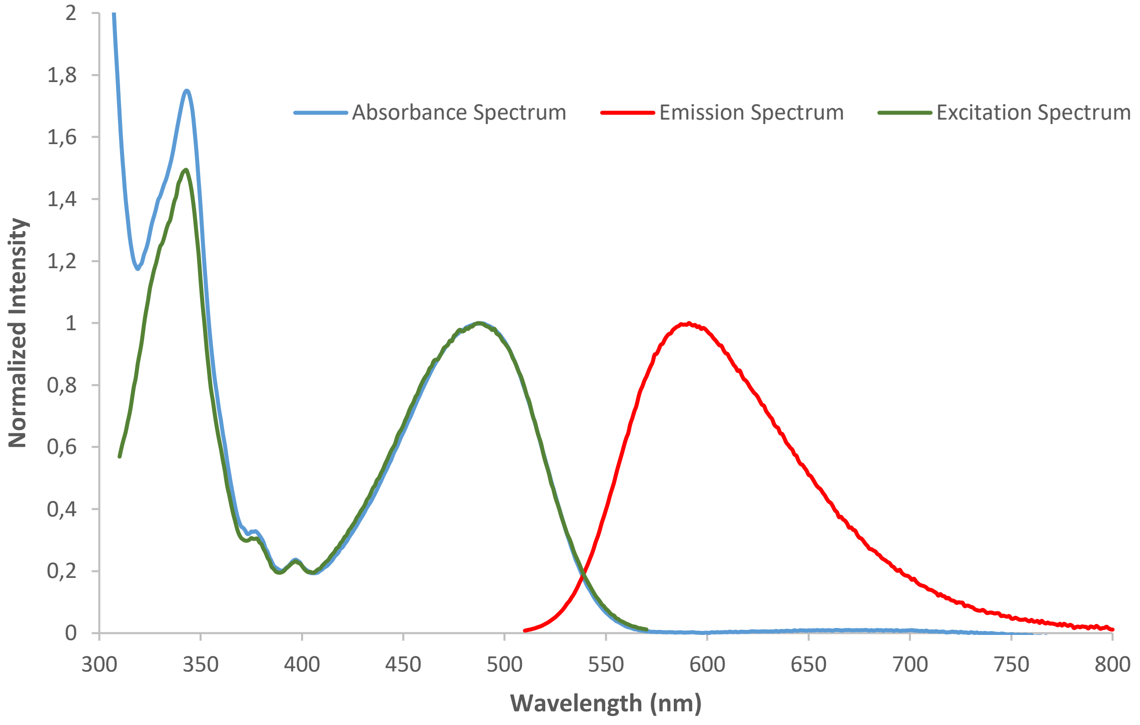

Fig S11: Spectral properties of CinNapht 5a in Ethanol:

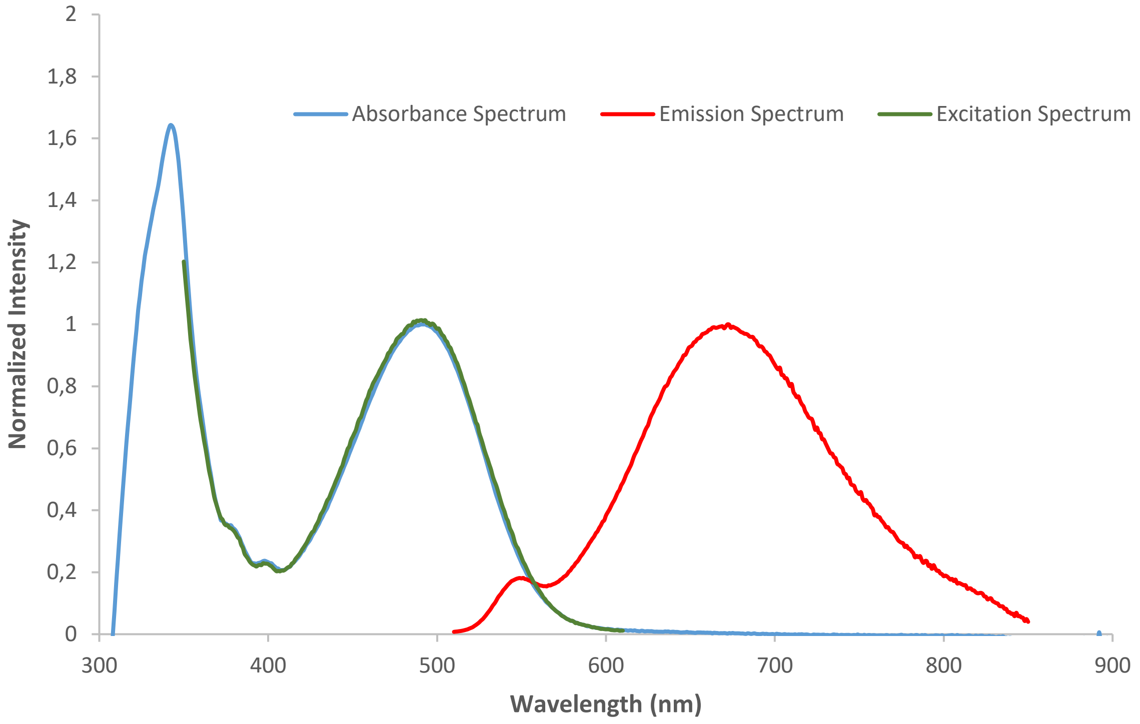

Fig S12: Spectral properties of CinNapht 5a in Methanol:

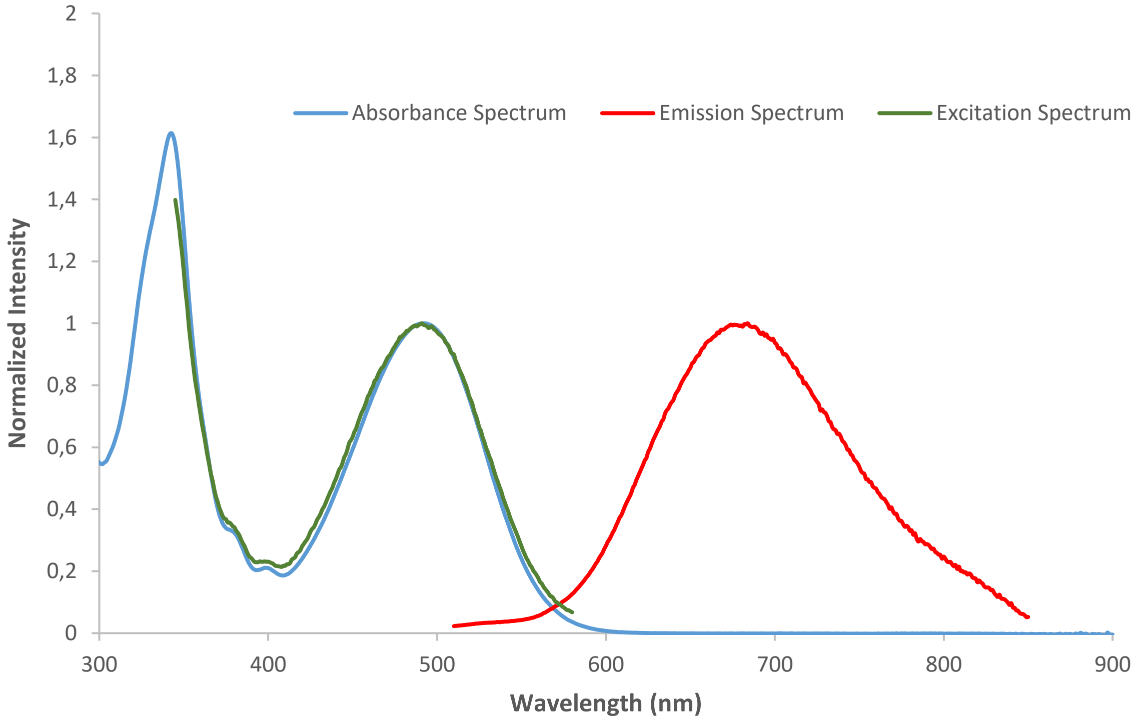

Fig S13: Spectral properties of CinNapht 5a in DMSO:

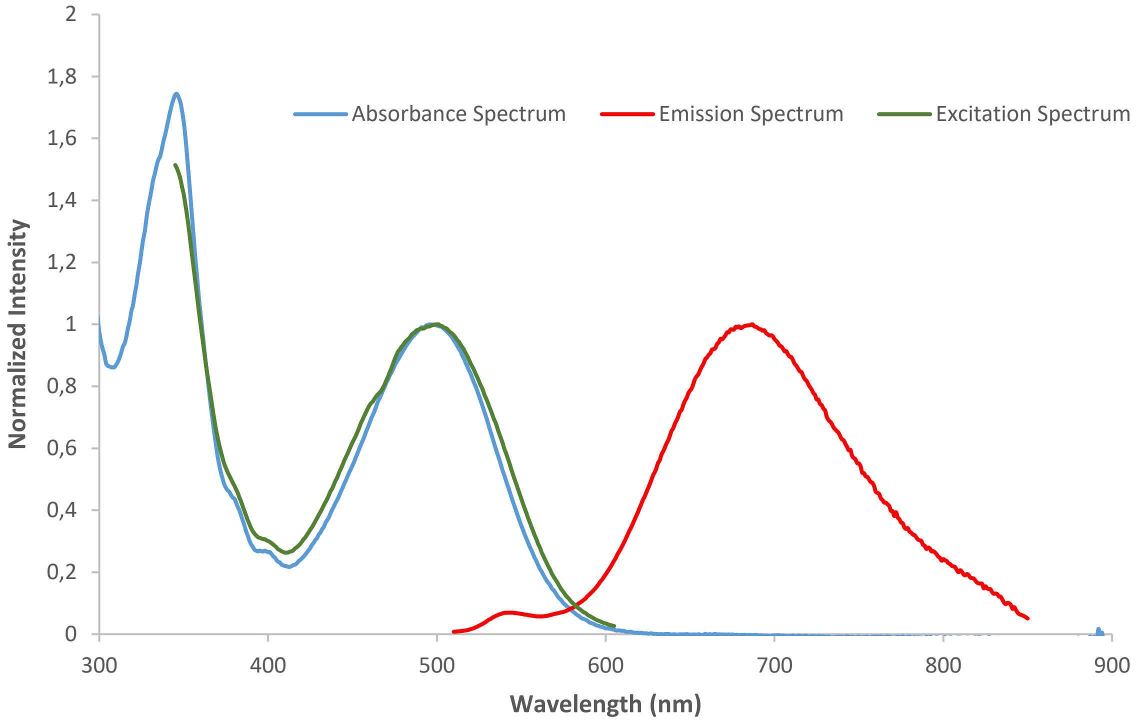

Fig S14: Spectral properties of CinNapht 5a in RPMI medium + 5% DMSO:

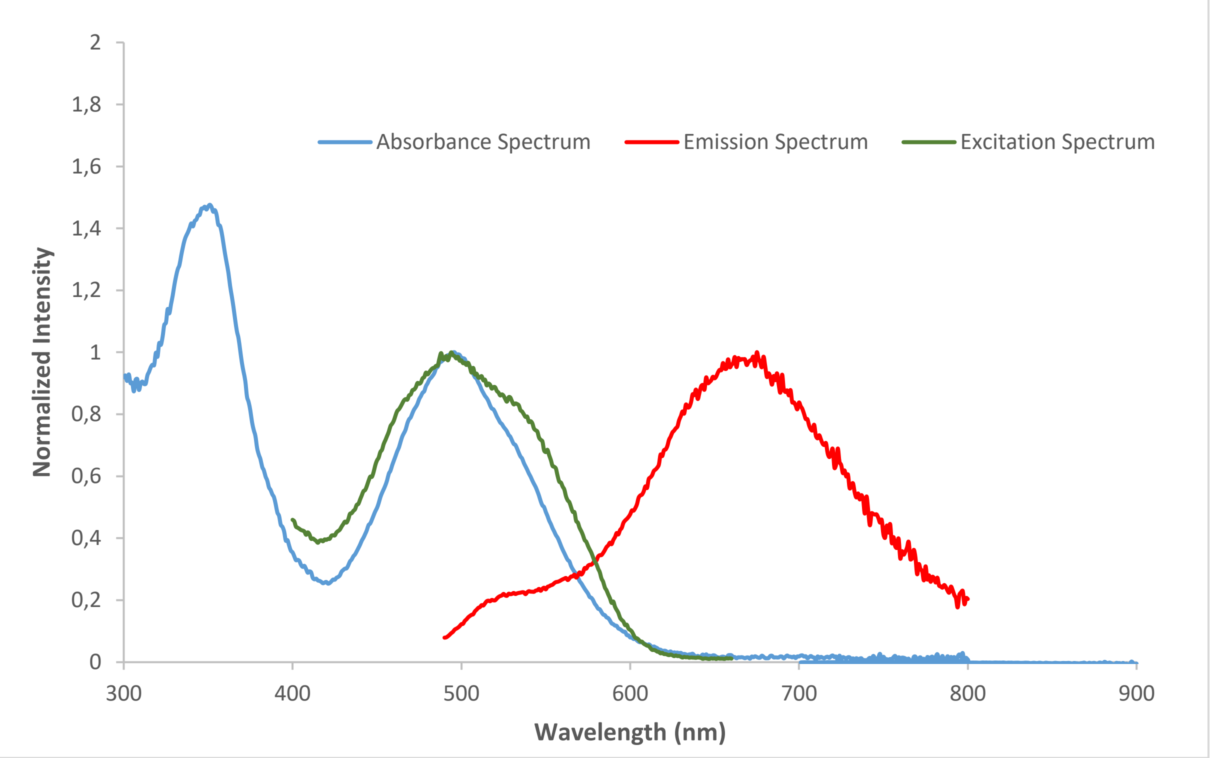

Fig S15: Spectral properties of CinNapht 5a in RPMI medium + 5% DMSO + 10 % FBS:

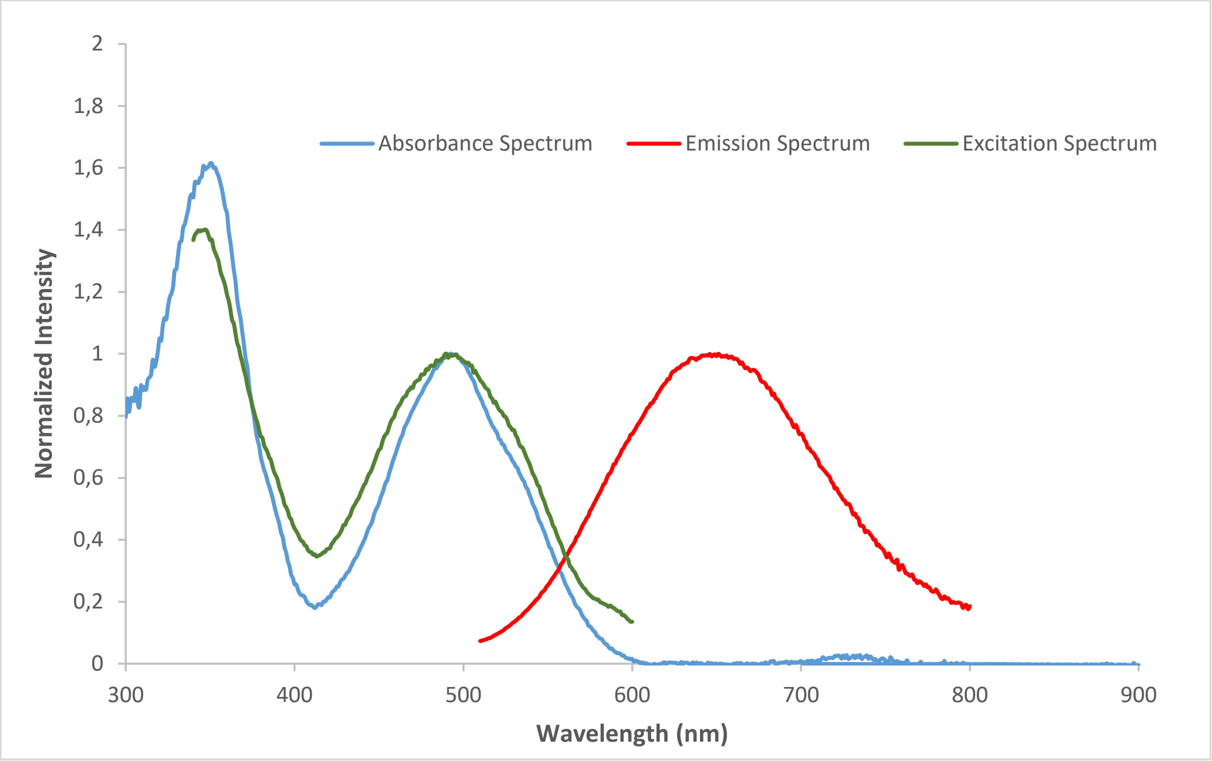

Fig S16: Superposition of normalized emission spectra of Naphthalimide and CinNapht 5a in DCM

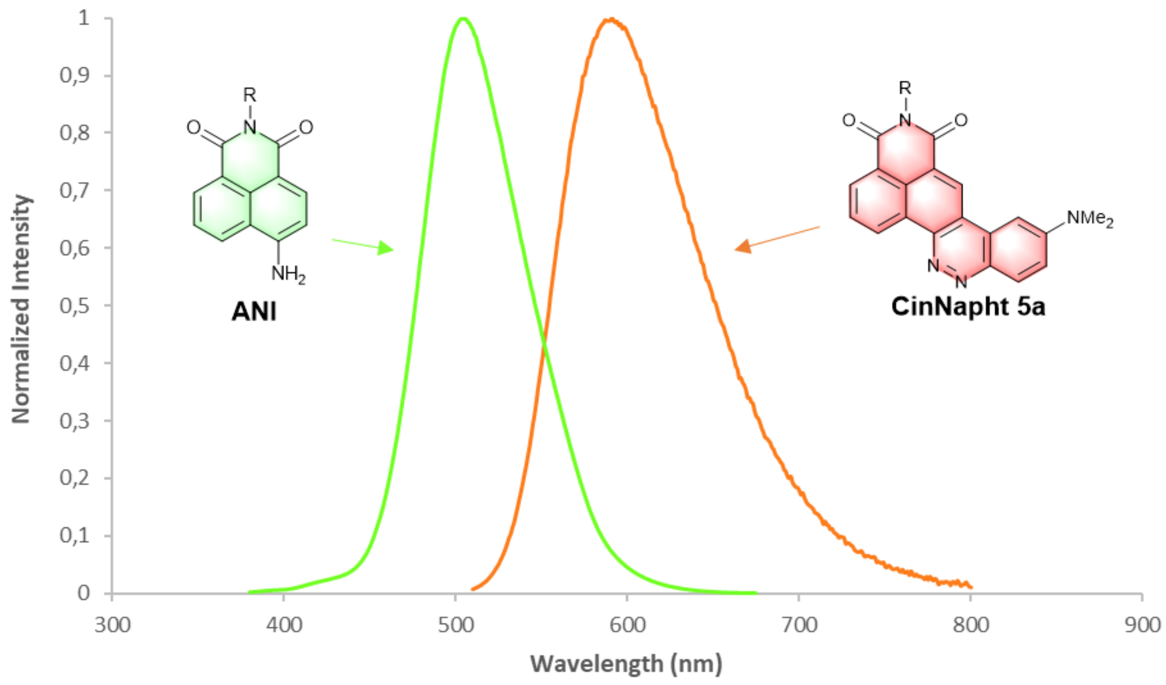

Fig S17: Emission spectra obtained with excitations at 343 nm and 488 nm in chloroform and Excitation spectrum for an emission centered at 585 nm

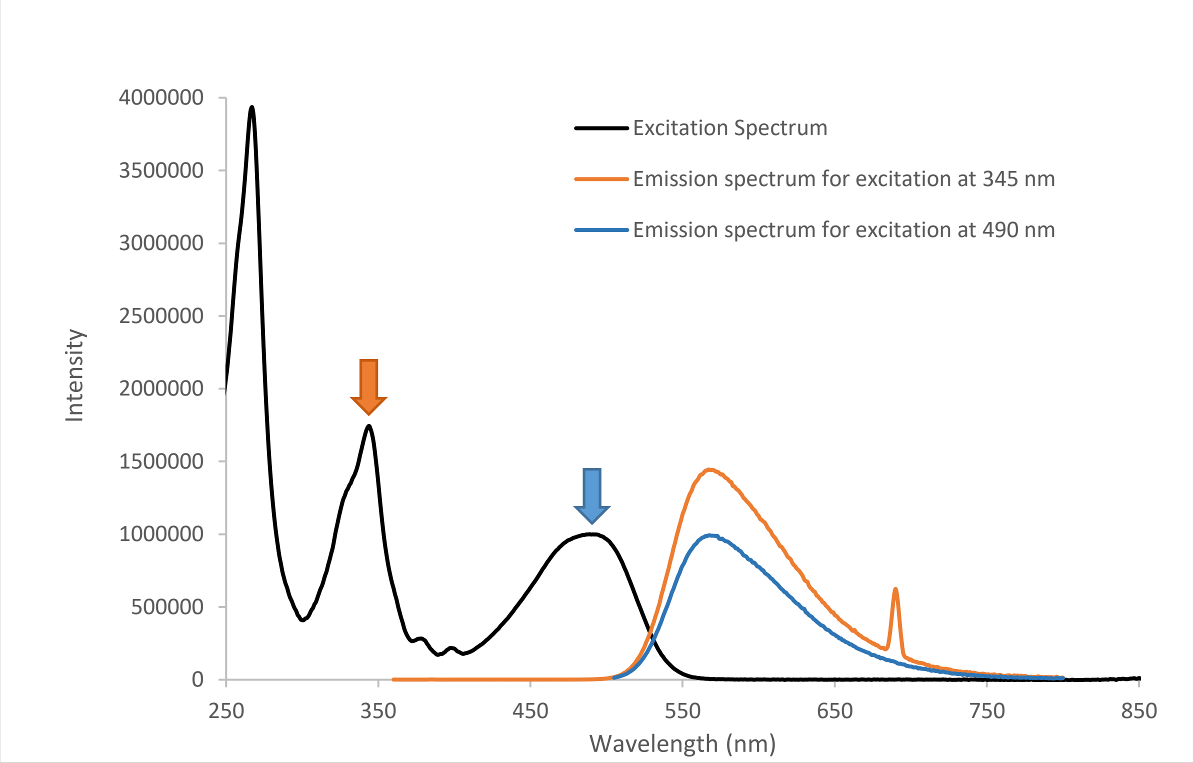

Fig S18: Solid State fluorescent properties of CinNapht 5a

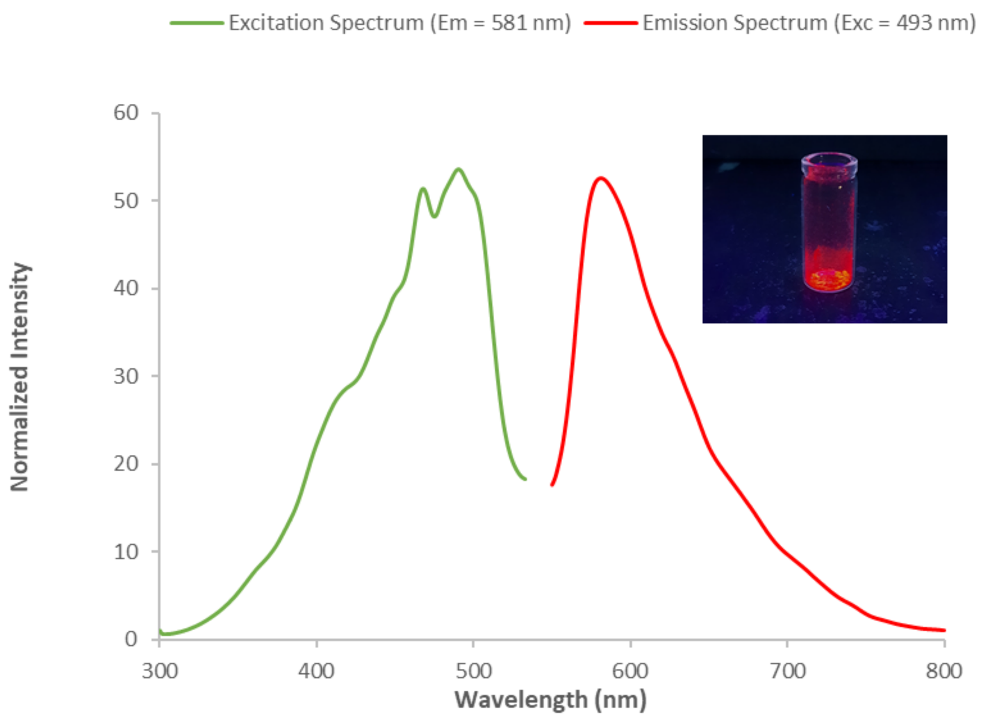

Fig S19: Correlation between Maximum emission wavelength and corresponding solvent polarity coefficient $E_T(30)$

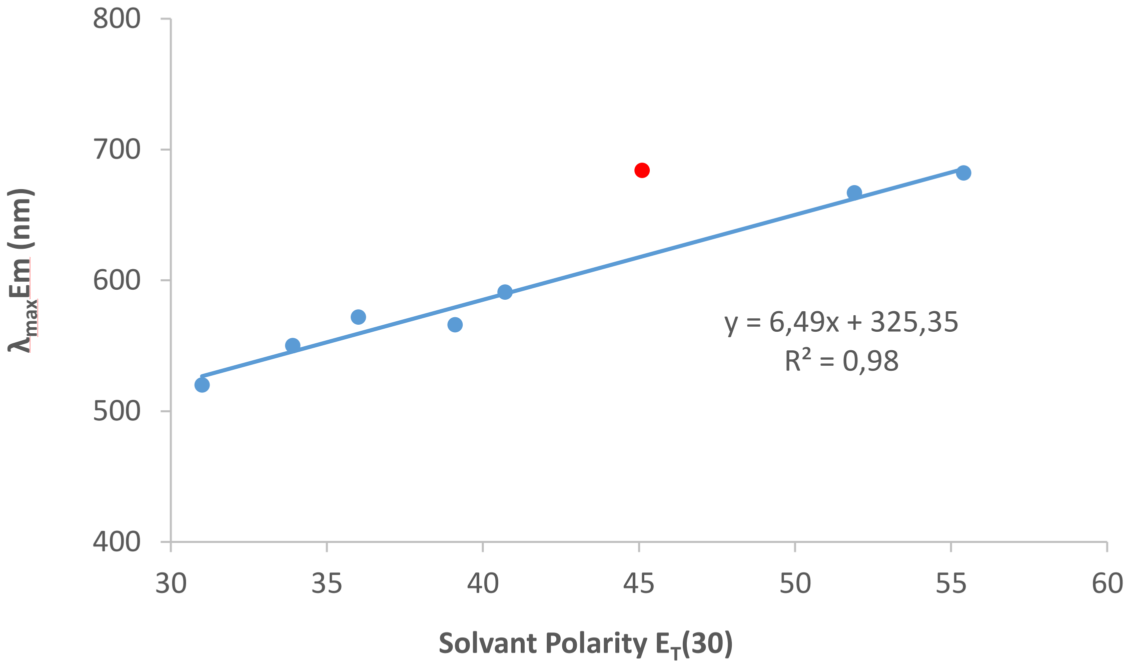

Fig S20: Table of complete values relative to Catalan method calculations[1]

Solvent	λ_{fluo} (nm)	$\bar{\nu}$ (cm^{-1})	$\bar{\nu}_0$ (cm^{-1})	a	SP	b	SdP	c	SA	d	SB
**n-hexane**	520	19231	19881	-767	0.616	-2506	0.000	-1506	0.000	-3182	0.056
**Toluene**	550	18182	19887	-774	0.782	-2523	0.284	-1506	0.000	-2998	0.128
**Dioxane**	572	17483	20046	-654	0.737	-2475	0.312	-1506	0.000	-2947	0.444
**Chloroform**	566	17668	20003	-683	0.783	-2472	0.614	-1503	0.047	-2986	0.071
**DCM**	591	16920	19968	-711	0.761	-2491	0.769	-1505	0.040	-2987	0.178
**DMSO**	682	14663	19905	-762	0.830	-2549	1.000	-1509	0.072	-3018	0.647
**Ethanol**	667	14993	19962	-717	0.633	-2495	0.783	-1501	0.400	-2982	0.658
**MeOH**	681	14684	19943	-729	0.608	-2511	0.904	-1510	0.605	-2994	0.545
**Mean value**			19950	-725		-2503		-1506		-3012	
**standard deviation**			54	40		24		3		67	

Fig S21: TD(DFT calculation)

Calculated electron density changes accompanying the first electronic excitation (the blue and red lobes signify decreases and increases respectively in electron density accompanying the electronic transition; their areas indicate the magnitude of the electron density change)[2]

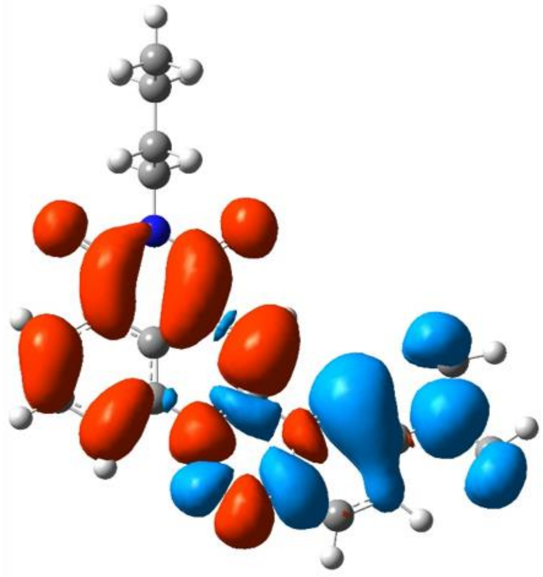

Fig S22: Excitation and Emission Spectra in living cell

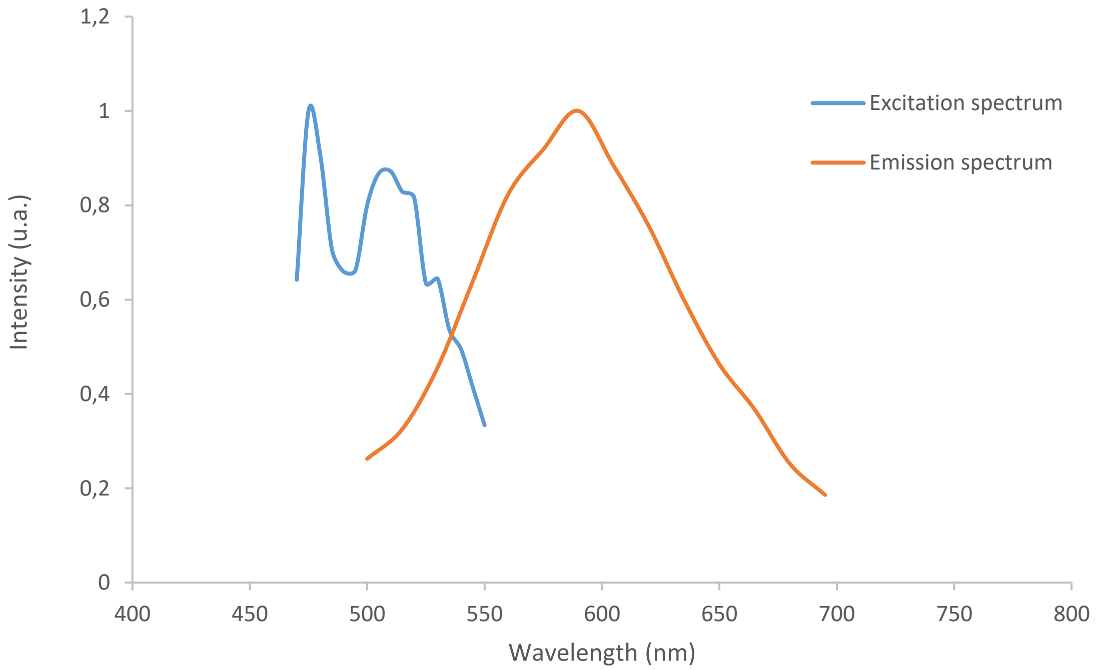

Fig S23: Cytotoxicity Study

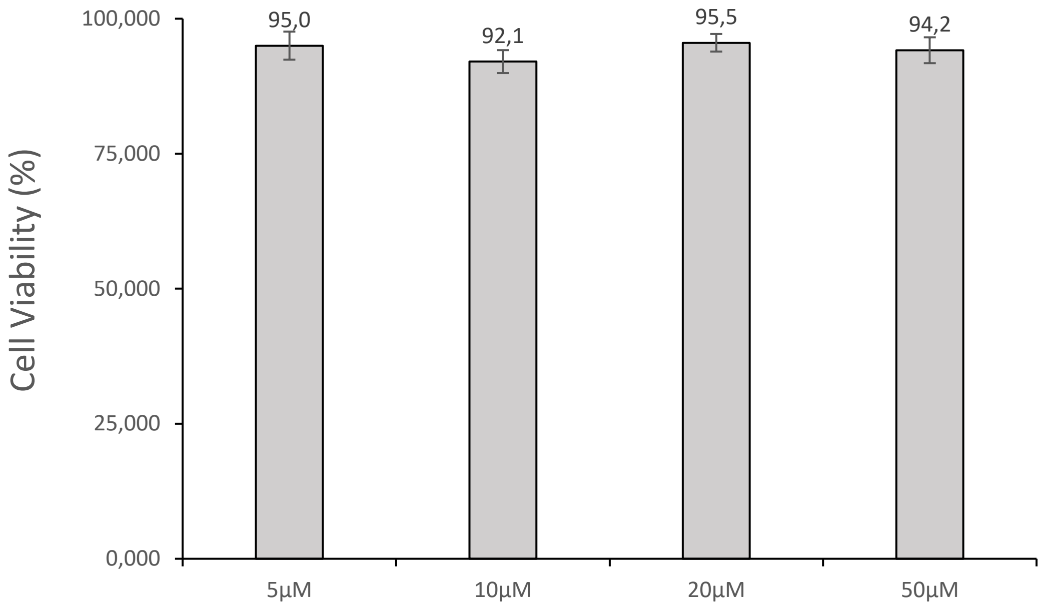

II. Abbreviations

The following abbreviations are used throughout the text of the ESI file: Abs, absorption; aq., aqueous; Ar, argon; DAD, diode array detector/detection; DCM, dichloromethane; DMF, Dimethylformamide; DMSO, dimethylsulfoxide; Em., emission; EtOAc, ethyl acetate; EtOH, Ethanol; Et_2O, Diethyl Ether; Ex., excitation; FA, formic acid; FLD, fluorescence detector; FT, Fourier transform; H_2O, water; Hept, Heptane; HFIP, Hexafluorophopanol; HPLC, high-pressure liquid chromatography; HRMS, high-resolution mass spectrum; IR, infrared; MeCN, acetonitrile; MeOH, methanol; min, minutes; NMR, nuclear magnetic resonance; PBS, phosphate buffered saline; PFA, paraformaldehyde; MS, mass spectrometry; PLC, preparative layer plate; RP, reversed phase; RPMI, Roswell park memorial institute medium; RT, room temperature; TFA, trifluoroacetic acid; THF, Tetrahydrofuran; TLC, Thin Layer Chromatography, t_R, retention time; UV, ultraviolet; vis, visible.

III. Experimental section

General

Unless otherwise noted, all commercially available reagents and solvents were used without further purification. TLC were carried out on silica gel aluminum plates with F-254 indicator; The spots were directly visualized or through illumination with UV lamp (λ = 254/365 nm). Flash-column chromatography purifications were performed on silica gel (40-63 µm) from Macherey-Nagel. Organic solvents for spectroscopy were purchased from Acros Organics or Sigma Aldrich. Absolute EtOH was provided by Carlo Erba. The HPLC grade MeCN used for RP-HPLC analyses was obtained from Carlo Erba. Formic acid (FA, puriss p.a., ACS reagent, reag. Ph. Eur., ≥98%) was provided by Merck-Millipore (brand Sigma-Aldrich). Aq. mobile-phases for HPLC were prepared using water purified with a Milli-Q Integral 3 system from Merck-Millipore (purified to 18.2 MW.cm).

Instrument and methods

[1]Proton NMR (^1H) spectra were recorded on a Bruker Avance 500 or 300 MHz and proton-decoupled carbon 13 NMR spectra were recorded at 125 MHz. NMR experiments were carried out in $CDCl_3$ or DMSO-*d*6 and chemical shifts are expressed in parts per million (ppm) from the residual non-deuterated solvent signal. Calibration was made by using residual signals of partially deuterated solvent summarized in 2010 by Fulmer et al.[3] The following abbreviations are used for the multiplicities: s: singlet; d: doublet; t: triplet; q: quadruplet; qt: quintuplet; m: multiplet or overlap of nonequivalent resonances; br s: broad singlet; Coupling constants (J) are reported in hertz (Hz). High resolution mass spectra were determined on an AEI MS-9 using electrospray ionization (ESI) and a time-offlight (TOF) analyzer. IR spectra were recorded with a PerkinElmer Spectrum BX FT-IR spectrometer directly from the substance via attenuated total reflectance (ATR-IR) and bond vibration frequencies are expressed in reciprocal centimeters (cm^{-1}). HPLC-MS analyses were performed on an Alliance W2690 system (Waters, USA) Melting points were measured with using a Büchi B-540 melting point apparatus.

Spectroscopy Studies

UV-vis absorption measurements (scan mode) were conducted on a Shimadzu UV-visible spectrophotometer UV-2600 using rectangular 10 mm path length quartz cuvettes from Thuet, at 25 °C. Fluorescence spectroscopic studies (scan and kinetics modes) were performed with a Fluoromax-4 spectrofluorometer (Jobin-Yvon; Horiba) at 25 °C, with rectangular 10 mm path length quartz cuvettes from Thuet. The absorption spectra of isoA were recorded with concentrations in the range 47-2.4 µM (total volume = 10 mL volumetric flask, six distinct dilutions for the accurate determination of molar extinction coefficients). For all the solvents, dilution were made from a stock solution in chloroform at 0.477 mM. The emission and excitation spectra were recorded with a diluted solution with 1% in volume of chloroform and lower than 0.1 absorbance values. For the excitation and emission spectra the slits width were adjusted between 2-5 nm, integration time = 0.1 s, 1 nm step, HV (S1) = 950 V). For chloroform, dichloromethane, ethanol, methanol and DMSO the excitation were performed at 488nm, at 478 nm for toluene, at 437 nm for n-hexane and at 470 nm for DMSO. For the excitation spectra, the emission was observed at 590 nm for DCM and $CHCl_3$, at 427 nm for ethanol, at 577 nm for toluene, at 600 nm for methanol and DMSO, at 518 nm for hexane and for dioxane. All fluorescence spectra were corrected from the lamp fluctuations and the apparatus response fluctuations. Fluorescence quantum yields were measured at 25 °C, using three diluted solution of isoA in the corresponding solvent (A<0.1) and three diluted solution of $Ru(bpy)_3^{2+}$ in air-saturated miliQ water (A<0.1). For each QY measurements, slidth width, excitation wavelength, scan rate, integration time and emission range were keep the same for the reference and the sample. The fluorescence quantum yield was determined thanks to the following formula:

$$\Phi_F(x) = Sx/Ss(n_X/n_S)^2\Phi_F(s)$$

Where S is the slope of the linear plot of the integrated fluorescence intensity in function of the absorbance value, n is the refractive index of the solvents (at 25 °C) used in measurements, and the subscripts s and x represent standard and unknown, respectively.

The fluorescence quantum yield for the reference, $Ru(bpy)_3^{2+}$ in air-saturated miliQ water, was measured to be 0.040 in aerated water with the Horiba K-sphere accessory, in good correlation with the literature values.[4]

For solid-state fluorescence, a solution of isoA in DCM was drop-casted on a microscope slide. The emission spectrum was then measured on a XENIUS, SAFAS fluorimeter. The excitation wavelength was fixed at 493nm, the band-width is 10 nm in excitation and emission, integration time = 0.1 s, 1 nm step.

Luminescence lifetime

Lifetime experiments were done using 10 mm path length quartz cuvette and a Fluoromax-4 spectrofluorometer (Jobin-Yvon; Horiba) equipped with a Horiba Scientific Nanoled source N485L. For the excitation we used a LED at 483 nm (950 V) with a pulse duration <200 ps. For MeOH, DCM and $CHCl_3$, the excitation was done with a 370 nm (950 V) Nanoled-370, pulse duration 1,3 ns. Each measurement was made with a pre-set peak of 12 000 hits and the emission was recorded at the fluorescence maximum. The prompt of the LED was recorded using a Ludox® HS-40 colloidal silica from Sigma Aldrich, a solution composed of silica nanoparticles (40% weight) in water, to diffuse the incident light. The experimental decay were fitted with a single or a biexponantial function (for MeOH, Dixoane, and DMSO), and the non-linear least-squares fit was obtained thanks to the Levenberg–Marquardt

algorithm. The fit quality was estimated thanks the calculated χ^2 (The variance of weigh residuals). The fit were estimate relevant for χ^2 values lower than 1.2.

In case of bi-exponential fit the average lifetime was determined using:

$$\bar{\tau} = \frac{B_1 T_1^2 + B_2 T_2^2}{B_1 T_1 + B_2 T_2}$$

with B the pre-exponential factor and T the lifetime of the fit.

High-performance liquid chromatography separations

Two chromatographic systems were used for the analytical experiments and the purification steps:

System A: Analysis were performed on an Alliance (W2690, Waters, USA) using Sunfire C18 column (5 µm 4.6 x 150 mm). Gradient was set up using (MeCN + 0.1 % Formic Acid) and 0.1% aq. FA eluents [5% MeCN (2.5 min), followed by linear gradient from 0% to 100% (15 min) of MeCN, then 100% MeCN + 0.1% FA for 5 min] at a flow rate of 1 mL/min. UV-Vis detection was carried out using a W2996 detector (Waters, USA) and "Max Plot" was used mode (220-795 nm). MS detection in ESI mode recorded using a SQD2 (Waters, USA). Fluorescence detection using a FLR2475 detector (Waters, USA) in 3D mode (Excitation at 500 nm and emission recorded between 510 and 800 nm

System B: Analysis were performed on an Alliance (W2690, Waters, USA) using Sunfire C18 column (5 µm 4.6 x 150 mm). Gradient was set up using (MeCN + 0.1 % Formic Acid) and 0.1% aq. FA eluents [5% MeCN (2.5 min), followed by linear gradient from 0% to 100% (20 min) of MeCN, then 100% MeCN + 0.1% FA for 5 min] at a flow rate of 1 mL/min. UV-Vis detection was carried out using a W2996 detector (Waters, USA) and "Max Plot" was used mode (240-795 nm). MS detection in ESI mode recorded using a SQD2 (Waters, USA).

Quantum chemical calculations

Ground state geometries were optimized at the B3LYP/6-31+g(d) level of calculation followed by a frequency calculation to confirm the convergence to a local minimum. A TDDFT calculation was done at the PBE0/6-311+g(d,p) level of theory. Then first singlet excited state geometries was optimized at the PBE0/6-311+g(d,p) level followed by a frequency calculation at the same level. Finally, a TDDFT calculation was done on this S1 optimized geometries at the PBE0/6-311+g(d,p) level of theory. Alternatively the N,N-dimethylamino substituent was twisted to 90°C and the TICT state optimized in its first excited state at the PBE0/6-311+g(d,p) level of theory followed by a frequency calculation. All calculations were done with Gaussian 16 (Revision B.01) software. Data were analyzed with GaussView 6.0 software.

Cell culture

Human A549 cancer cell line was obtained from the American type Culture Collection (Rockville, USA) and was grown in RPMI 1640 supplemented with 10% fetal calf serum (FCS) and 1% glutamine according to the supplier's instructions. 2.10^4 A459 cells were plated in Lab-Teck® chambers and after 48 hours of incubation at 37 °C in a humidified atmosphere containing 5% CO_2, the medium was removed and replaced by fresh RPMI 1640 containing the fluorophore at 5 µM final concentration. The cells were incubated for 2 h at 37 °C in a humidified atmosphere containing 5% CO_2. The fluorophore solution was then removed and the cells were washed thrice with PBS at RT. A 4% solution of PFA in PBS was then added and the cells were incubated at RT for 15 min until fixation. After removing the PFA solution, cells were washed thrice with PBS (1X) then prepared for microscopy.

Fluorescence microscopy

Once cell fixation was achieved, the culture chambers were removed and the microscope slide was prepared using a Fluoroshield mounting medium. Fluorescence images were acquired using a Leica SP8-X inverted confocal microscope with a 40× oil immersion objective (HC PL APO CS2 Leica). Excitation was performed using a White laser pulsed at 80MHz and excitation was applied at 475 nm. Detection was carried out by using PMT detector (Hamamatsu 6357) collecting photons from 500 nm to 700 nm. Recorded image size was 1024x1024 pixels and a pixel size of 65nm was reach using the zoom factor according sampling laws.

Cytotoxicity Study

Cell viability was determined by a luminescent assay according to the manufacturer's instructions (Promega, Madison, WI, USA). Briefly, A549 cells were seeded in 96-well plates (2.5 × 103 cells/well) containing 90 µL of growth medium. After 24 h of culture, the cells were treated with the tested compounds at 5, 10, 20 and 50 µM final concentrations. Control cells were treated with the vehicle. After 72 h of incubation, 100 µL of CellTiter Glo Reagent was added for 15 min before recording luminescence with a spectrophotometric plate reader PolarStar Omega (BMG LabTech). The percent viability index was calculated from three experiments.

Synthesis protocols

6-amino-1H,3H-benzo[de]isochromene-1,3-dione (1)

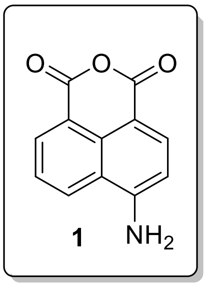

$C_{12}H_7NO_3$
MW: 213.19 g.mol^{-1}
Yield: 76%
Yellow-O solid

To a suspension of 4-bromo-1,8-naphthalic anhydride (5.00 g, 18.0 mmol, 1 eq.) in DMF (100 mL) was added sodium azide (1.76 g, 27.0 mmol, 1.5 eq.). The resulting reaction mixture was heated at 60 °C for 2 h. After cooling to room temperature, the yellow brown solution was diluted with cold H_2O, and the suspension was filtered. The residue was washed with water and directly used for the next step. The crude azide intermediate was suspended in a mixture of THF (250 mL) and 0.5 M HCl aq (50 mL), and then triphenylphosphine (4.71 g, 18.0 mmol, 1 eq.) was slowly added to the suspension with stirring. After stirring at room temperature for 30 min, the mixture was basified with 1M NaOH aq (34 mL) and evaporated under reduced pressure to remove THF. The resulting mixture was diluted with AcOEt (70 mL) and filtered to afford the crude product as a solid. The product was washed with EtOAc and water, and dried *in vacuo* to afford compound **1** (2.9 g, 76%). **mp** > 390 °C; **Rf** (DCM/MeOH, 90:10, v/v) = 0.65; **^{1}H NMR** (500 MHz, DMSO-*d*6) δ 8.66 (d, *J* = 8.4 Hz, 1H), 8.39 (d, *J* = 7.3 Hz, 1H), 8.15 (d, *J* = 8.5 Hz, 1H), 7.76 (s, 2H), 7.65 (m, 1H), 6.85 (d, *J* = 8.6 Hz, 1H); **^{13}C NMR** (126 MHz, DMSO-*d*6) δ 162.0, 160.3, 153.9, 135.8, 132.9, 132.5, 130.7, 124.3, 119.3, 118.2, 108.7, 102.2; **IR** (neat): ν = 3428, 3337, 3232, 1727, 1687, 1647, 1620, 1570, 1524, 1479, 1405, 1387, 1358, 1308, 1227, 1175, 1162, 1107, 1006, 976, 889, 850, 810, 767, 749, 728, 677 cm^{-1}; **HRMS** calculated for $C_{12}H_7NO_3$ $[M+H]^+$ 214.0504, found 214.0503

***6-amino-5-bromo-1H,3H-benzo[de]isochromene-1,3-dione* (2)**

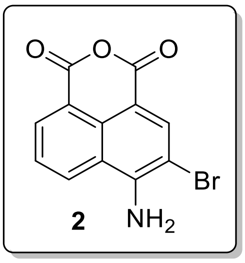

$C_{12}H_6BrNO_3$
MW: 292.09 g.mol^{-1}
Yield: 92%
Yellow solid

To a solution of compound **1** (500 mg, 2.35 mmol, 1 eq.) in HFIP (15 mL) was added *N*-bromosuccinimide (461 mg, 2.59 mmol, 1.1 eq.) slowly at room temperature.[5] The reaction mixture was stirred for 2 hours before removing solvent under reduced pressure. The residue was taken in $CHCl_3$ (2 mL) and the mixture was heated at reflux and then quickly filtered. The solid was washed with hot $CHCl_3$ to afford compound **2** (630 mg, 92%) as a yellow solid. **mp** 330 °C; **Rf** (DCM/MeOH, 90:10, v/v) = 0.8; **^{1}H NMR** (500 MHz, DMSO-*d*6) δ 8.82 (d, *J* = 8.5 Hz, 1H), 8.44 (d, *J* = 7.2 Hz, 1H), 8.33 (s, 1H), 7.85 – 7.64 (m, 3H); **^{13}C NMR** (126 MHz, DMSO-*d*6) δ 161.2, 159.4, 149.7, 137.4, 132.8, 130.9, 130.7, 125.6, 119.7, 118.6, 104.5, 101.5; **IR** (neat): ν = 3467, 3364, 3230, 1752, 1711, 1633, 1573, 1513, 1467, 1396, 1378, 1357, 1311, 1273, 1225, 1204, 1143, 1114, 1203, 993, 919, 824, 792, 769, 750, 699, 666 cm^{-1}; **ESI-HRMS** calculated for $C_{12}H_6BrNO_3$ $[M+H]^+$ 291.9609, found 291.9604

***6-amino-5-bromo-2-butyl-1H-benzo[de]isoquinoline-1,3(2H)-dione* (3)**

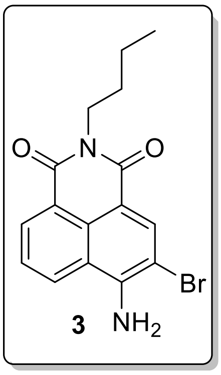

$C_{16}H_{15}BrN_2O_2$
MW: 347.21 g.mol^{-1}
Yield: 92%
Yellow solid

To a mixture of compound **2** (1.00 g, 3.42 mmol, 1 eq.) in absolute EtOH (20 mL) was added n-butylamine (0.68 mL, 6.84 mmol, 2 eq.). The reaction mixture was heated at 90°C overnight. The reaction mixture was then filtered and washed with Et_2O affording compound **3** (1.00 g, 92%) as a yellow solid. **mp** 195 °C; **Rf** (Hept/AcOEt, 50:50, v/v) = 0.5; **^{1}H NMR** (500 MHz, $CDCl_3$) δ 8.61 (s, 1H), 8.59 (d, *J* = 6.9 Hz, 1H), 8.09 (d, *J* = 8.0 Hz, 1H), 7.69 (t, *J* = 7.6 Hz, 1H), 5.43 (brs, 2H), 4.15 (t, *J* = 7.1 Hz, 2H), 1.78 – 1.63 (m, 2H), 1.43 (qt, *J* = 7.4 Hz, 2H), 0.97 (t, *J* = 7.4 Hz, 3H); **^{13}C NMR** (126 MHz, DMSO-*d*6) δ 163.3, 162.0, 148.5, 135.7, 130.9, 129.3, 128.2, 125.3, 122.0, 120.0, 109.4, 101.5, 39.1, 29.7, 19.8, 13.7; **IR** (neat): ν = 3638, 3562, 3448, 3329, 3231, 3063, 2954, 2869, 1704, 1623, 1623, 1572, 1509, 1461, 1431, 1392, 1364, 1348, 1332, 1273, 1226, 1166, 1141, 1117, 1079, 971, 936, 898, 838, 803, 773, 750, 737, 674, 665 cm^{-1}; **ESI-HRMS** calculated for $C_{16}H_{15}BrN_2O_2$ $[M+H]^+$ 347.0395, found 347.0389.

***6-amino-2-butyl-5-(3-(dimethylamino)phenyl)-1H-benzo[de]isoquinoline-1,3(2H)-dione* (4)**

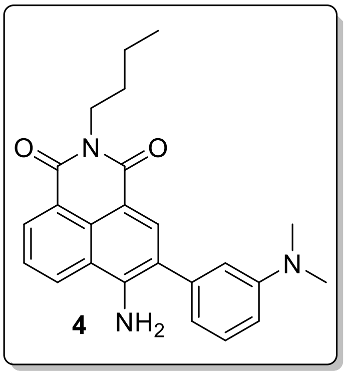

$C_{24}H_{25}N_3O_2$
MW: 387.48 g.mol^{-1}
Yield: 82%
Orange solid

Compound **3** (200 mg, 0.58 mmol, 1 eq.), (3-(dimethylamino)phenyl)boronic acid (106 mg, 0.64 mmol, 1.1 eq.), and Na_2CO_3 (184 mg, 1.74 mmol, 3 eq.) were dissolved in H_2O, EtOH, and toluene (3.20 mL, 3:3:10 v/v) in a Schlenk tube. The resulting mixture was deoxygenated with freeze-pump-thaw cycling before addition of Pd(dppf)Cl_2 (42.0 mg, 0.06 mmol, 0.1 eq.). The reaction was heated at 80 ˚C with constant stirring under argon for 17 h. The mixture was cooled to room temperature, it was diluted with DCM and washed with water. The organic layer was collected, dried over $MgSO_4$, and concentrated under reduced pressure. The residue was purified by flash chromatography on silica gel (Hept/AcOEt 9:1 to 8:2) to afford compounds **4** (183 mg, 82%) as an orange solid. **mp** 201 °C; **Rf** (Hept/AcOEt, 60:40, v/v) = 0.3; **^{1}H NMR** (500 MHz, $CDCl_3$) δ 8.58 (dd, *J* = 7.3, 1.0 Hz, 1H), 8.45 (s, 1H), 8.16 (dd, *J* = 8.4, 1.1 Hz, 1H), 7.64 (dd, *J* = 8.4, 7.3 Hz, 1H), 7.39 – 7.33 (m, 1H), 6.82 (dt, *J* = 7.3, 1.2 Hz, 1H), 6.80 – 6.76 (m, 2H), 5.22 (s, 2H), 4.21 – 4.13 (m, 2H), 3.00 (s, 6H), 1.77 – 1.66 (m, 2H), 1.44 (qt, *J* = 7.4 Hz, 2H), 0.97 (t, *J* = 7.4 Hz, 3H); **^{13}C NMR** (125 MHz, $CDCl_3$) δ 164.8, 164.4, 151.4, 146.1, 138.8, 135.1, 131.1, 130.1, 129.2, 127.3, 125.3, 123.7, 123.3, 120.5, 117.1, 113.3, 112.2, 111.8, 40.7, 40.2, 30.5, 20.6, 14.0; **IR** (neat): ν = 3482, 3370, 3236, 3064, 2960, 2928, 2862, 2802, 1677, 1625, 1614, 1574, 1518, 1497, 1425, 1391, 1371, 1335, 1288, 1226, 1179, 1135, 1077, 1063, 992, 959, 938, 926, 890, 850, 816, 791, 774, 745, 716, 692, 680 cm^{-1}; **ESI-HRMS** calculated for $C_{24}H_{25}N_3O_2$ $[M+H]^+$ 388.2025, found 388.2028.

***11-butyl-2-(dimethylamino)-10H-benzo[c]isoquinolino[4,5-gh]cinnoline-10,12(11H)-dione* (5a)**

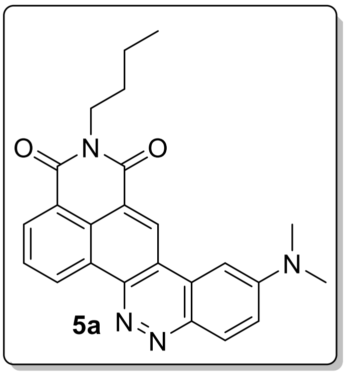

$C_{24}H_{22}N_4O_2$
MW: 398.47 g.mol^{-1}
Yield: 73%
Red solid

A solution of compound **4** (45.0 mg, 0.116 mmol, 1eq.) in CH_3CN was cooled in an ice bath to 0 °C. Nitrosyl tetrafluoroborate (15.0 mg, 0.128 mmol, 1.1 eq.) was added while maintaining the reaction mixture at 0 °C. The mixture was stirred for 1h before neutralizing with saturated aqueous $NaHCO_3$ solution and extracting with DCM. The organic layer was dried over $MgSO_4$ and concentrated under reduced pressure to give crude product, which was purified by flash chromatography on silica gel (DCM/AcOEt 8:2 to 2:1, v/v) to afford compound **5a** (34.0 mg, 73%) as a red solid. **mp** 320 °C (decomposition); **Rf** (DCM/AcOEt, 80:20, v/v) = 0.4; **^{1}H NMR** (500 MHz, $CDCl_3$) δ 9.88 (dd, *J* = 8.3, 1.2 Hz, 1H), 9.29 (s, 1H), 8.66 (dd, *J* = 7.5, 1.2 Hz, 1H), 8.45 (d, *J* = 9.2 Hz, 1H), 7.99 (t, *J* = 7.8 Hz, 1H), 7.37 (dd, *J* = 9.3, 2.6 Hz, 1H), 7.28 (d, *J* = 2.5 Hz, 1H), 4.24 – 4.18 (m, 2H), 3.28 (s, 6H), 1.79 – 1.72 (m, 2H), 1.50 (dt, *J* = 15.0, 7.4 Hz, 2H), 1.01 (t, *J* = 7.4 Hz, 3H); **^{13}C NMR** (126 MHz, $CDCl_3$) δ 164.2, 163.9, 152.3,

143.1, 142.0, 133.0, 131.3, 131.0, 129.8, 128.7, 127.5, 126.5, 124.3, 123.1, 122.6, 118.2, 97.3, 40.7, 40.6, 30.4, 20.6, 14.0; **IR** (neat): ν = 3067, 2949, 2929, 2871, 1698, 1658, 1599, 1525, 1493, 1447, 1428, 1394, 1377, 1367, 1329, 1311, 1289, 1269, 1243, 1223, 1186, 1132, 1117, 1065, 1008, 985, 939, 922, 905, 866, 824, 813, 790, 752, 743, 732, 719, 691, 666 cm^{-1}; **ESI-HRMS** calculated for $C_{24}H_{22}N_4O_2$ $[M+H]^+$ 399.1821, found 399.1819.

***11-butyl-4-(dimethylamino)-10H-benzo[c]isoquinolino[4,5-gh]cinnoline-10,12(11H)-dione* (5b)**

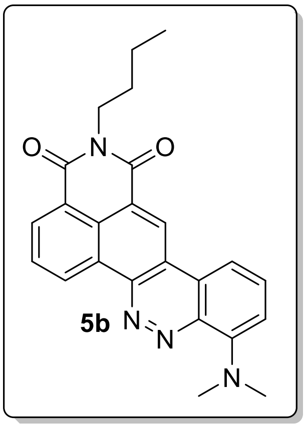

$C_{24}H_{22}N_4O_2$
MW: 398.47 $g.mol^{-1}$
Yield: 16%
Deep purple solid

A solution of $NaNO_2$ (8.90 mg, 0.13 mmol, 1eq.) in H_2O (1.35 mL) was added slowly to a stirred solution of compound **4** (50.0 mg, 0.13 mmol, 1eq.) in a dilute aqueous HCl solution (1M, 650 µL, 0.65 mmol, 5eq.) at 0 °C. CH_3CN (2.00 mL) was added to the reaction mixture for better dissolution of reagents. The solution was stirred for 4 hours before neutralizing with saturated aqueous $NaHCO_3$ solution and extracting with DCM. The organic layer was dried over $MgSO_4$ and concentrated under reduced pressure to give crude product, which was purified by flash chromatography on silica gel (DCM/AcOEt 8:2 to 2:1, v/v) to afford compound **5b** as a deep purple solid (8.40 mg, 16%) (note that compound **5a** was also isolated (30 mg, 58%)). **mp** 305 °C (decomposition); **Rf** (Hept/AcOEt, 50:50) = 0.6; **^{1}H NMR** (500 MHz, $CDCl_3$) δ 9.97 (dd, *J* = 8.2, 1.3 Hz, 1H), 9.52 (s, 1H), 8.73 (dd, *J* = 7.4, 1.2 Hz, 1H), 8.10 – 7.99 (m, 2H), 7.82 (t, *J* = 8.0 Hz, 1H), 7.19 (d, *J* = 8.0 Hz, 1H), 4.23 (dd, *J* = 8.6, 6.6 Hz, 2H), 3.46 (s, 6H), 1.80 – 1.73 (m, 2H), 1.49 (qt, *J* = 7.4 Hz, 2H), 1.01 (t, *J* = 7.4 Hz, 3H); **^{13}C NMR** (75 MHz, $CDCl_3$) δ 164.2, 163.6, 151.7, 141.6, 141.3, 133.7, 131.5, 130.7, 129.7, 129.0, 127.5, 126.9, 124.4, 123.4, 122.8, 119.5, 114.7, 110.9, 45.2, 40.7, 30.4, 20.6, 14.0; **IR** (neat): ν = 3676, 3076, 2957, 1981, 1933, 1698, 1652, 1618, 1592, 1552, 1501, 1428, 1406, 1360, 1346, 1328, 1309, 1228, 1188, 1154, 1106, 1059, 1042, 956, 937, 922, 862, 840, 816, 785, 769, 736, 697, 662 cm^{-1}; **ESI-HRMS** calculated for $C_{24}H_{22}N_4O_2$ $[M+H]^+$ 399.1821, found 399.1802.

IV. NMR and MS Spectra

^{1}H NMR of compound 1

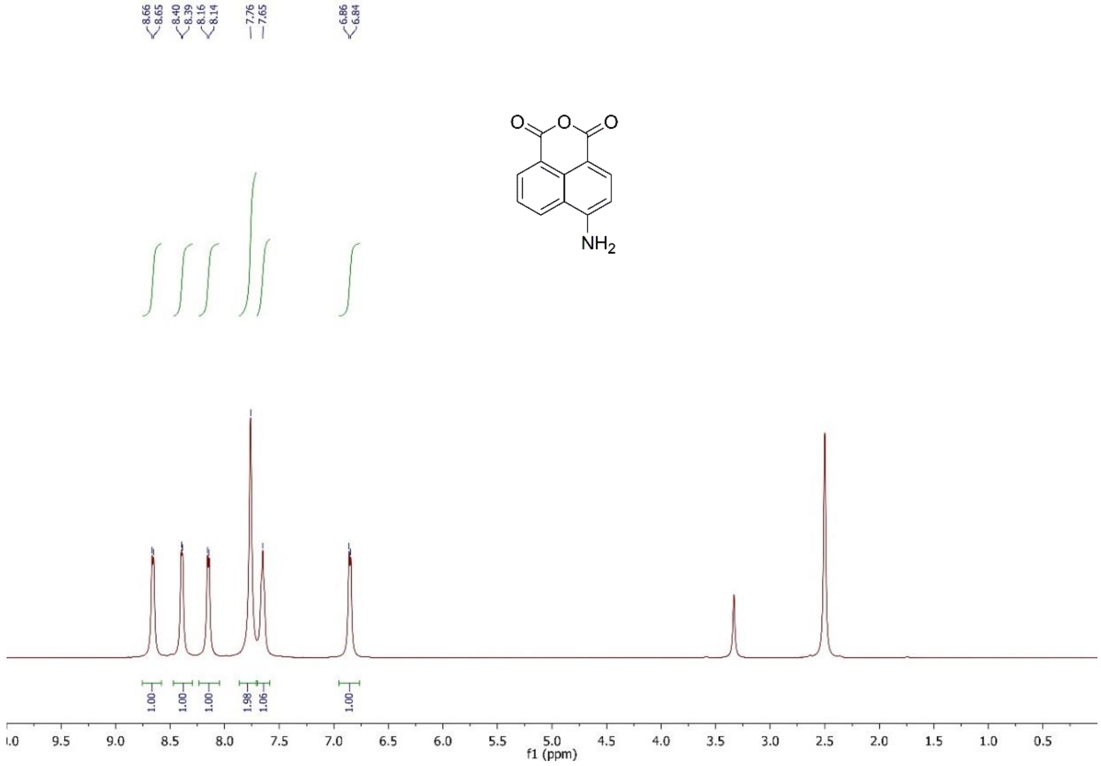

^{13}C NMR of compound 1

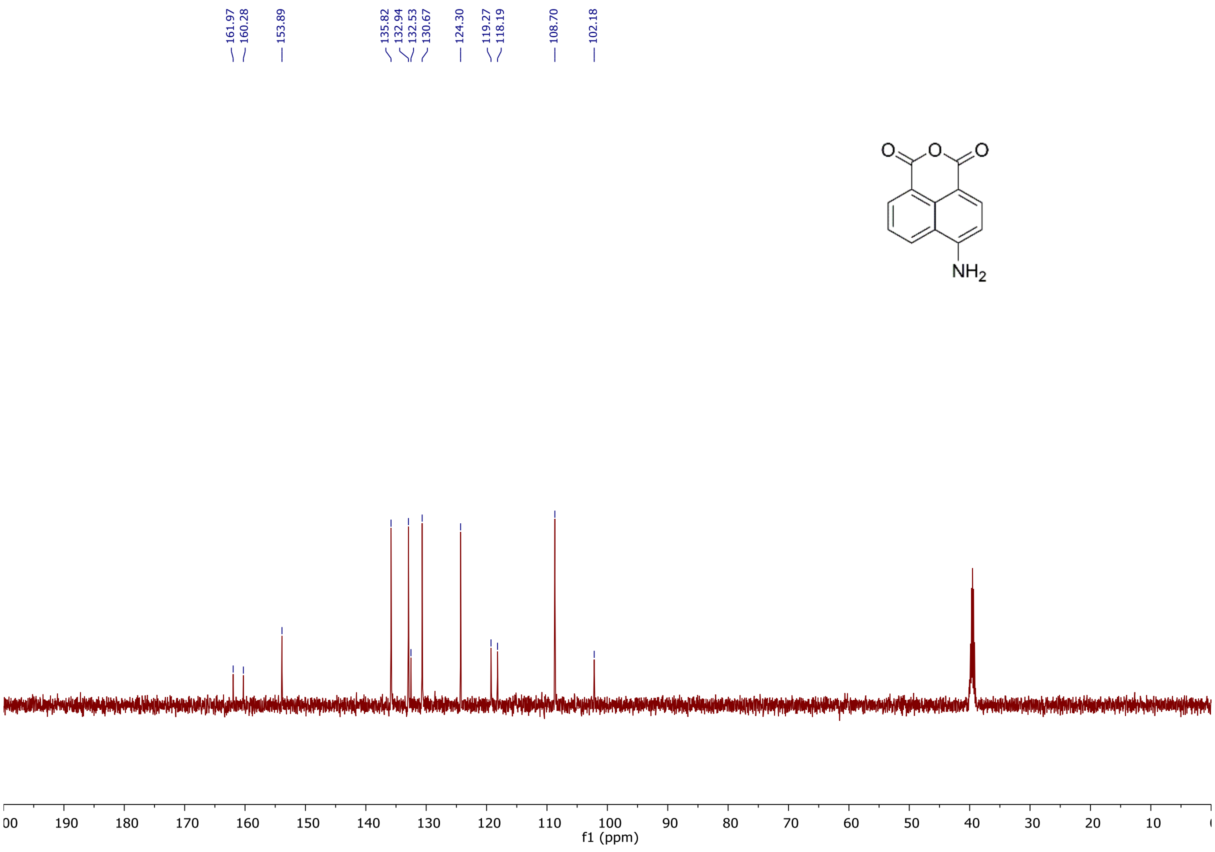

HRMS (ESI+) of compound 1

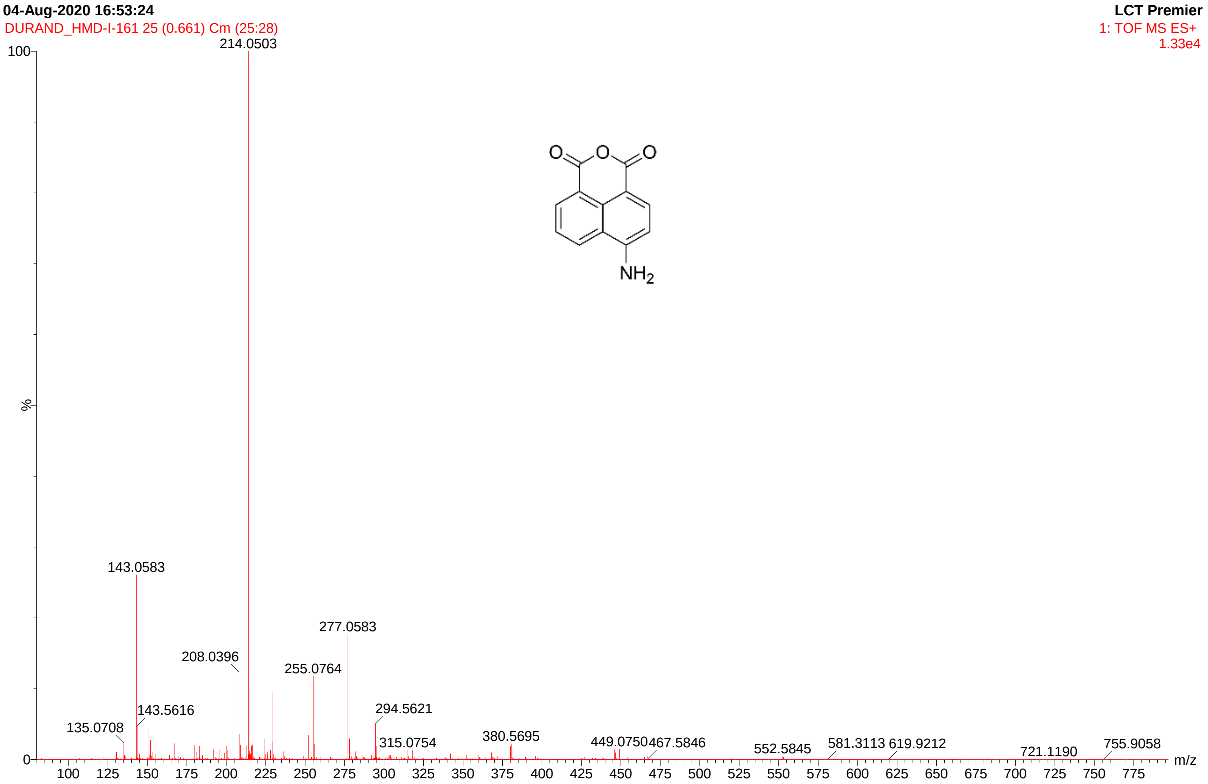

^{1}H NMR of compound 2

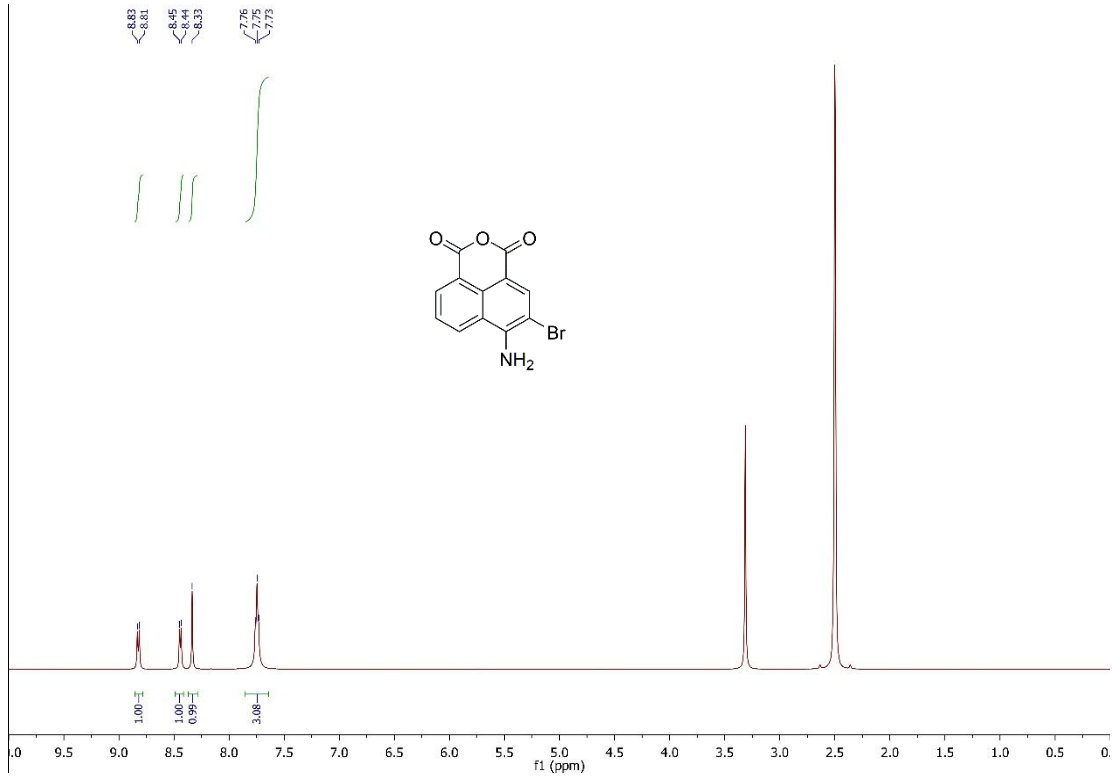

^{13}C NMR of compound 2

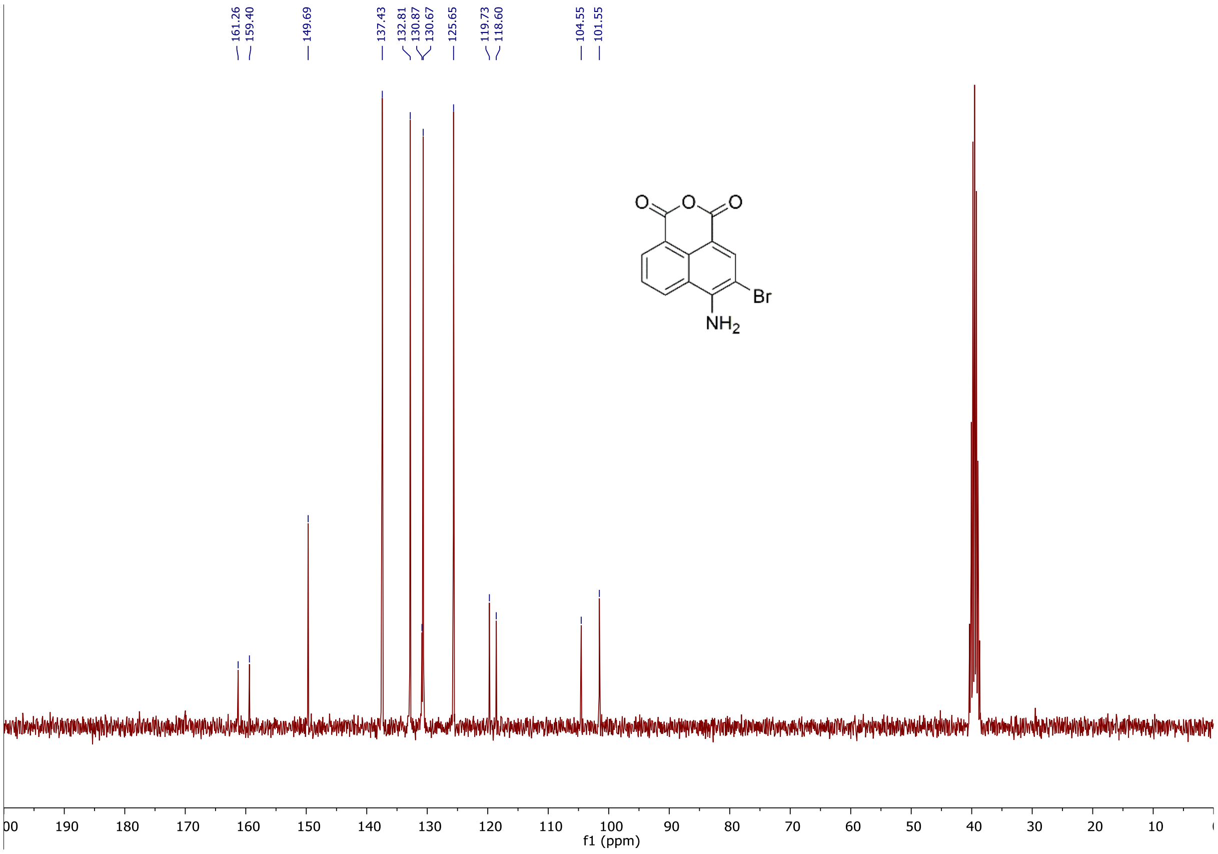

HRMS (ESI+) of compound 2

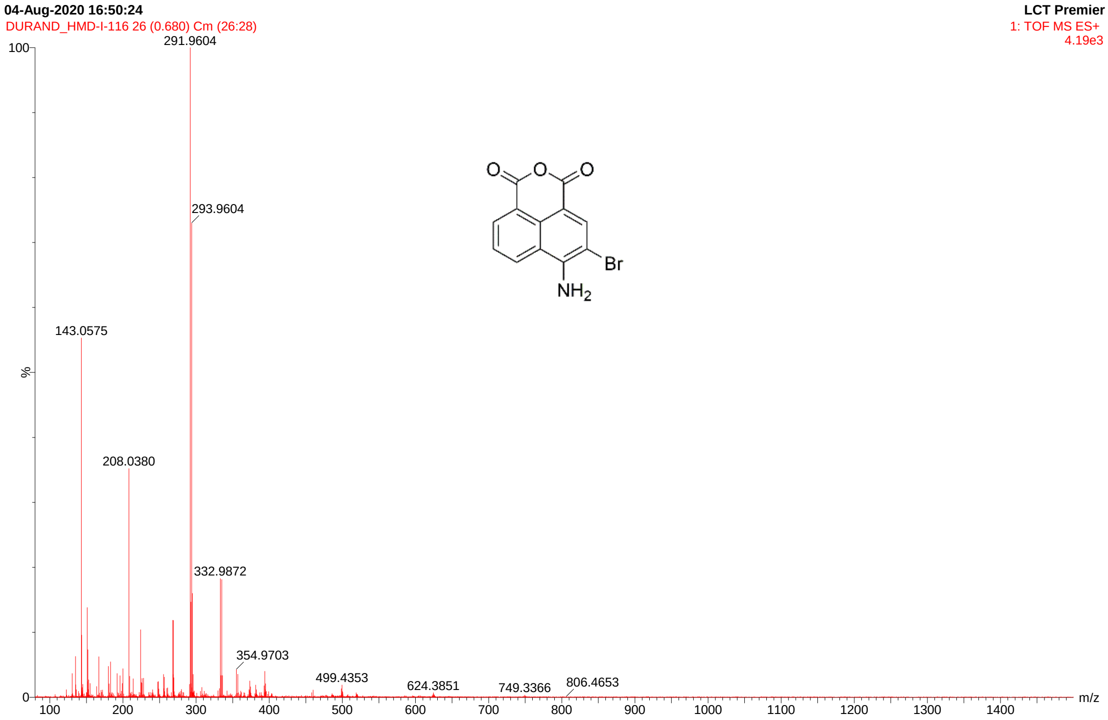

^{1}H NMR of compound 3

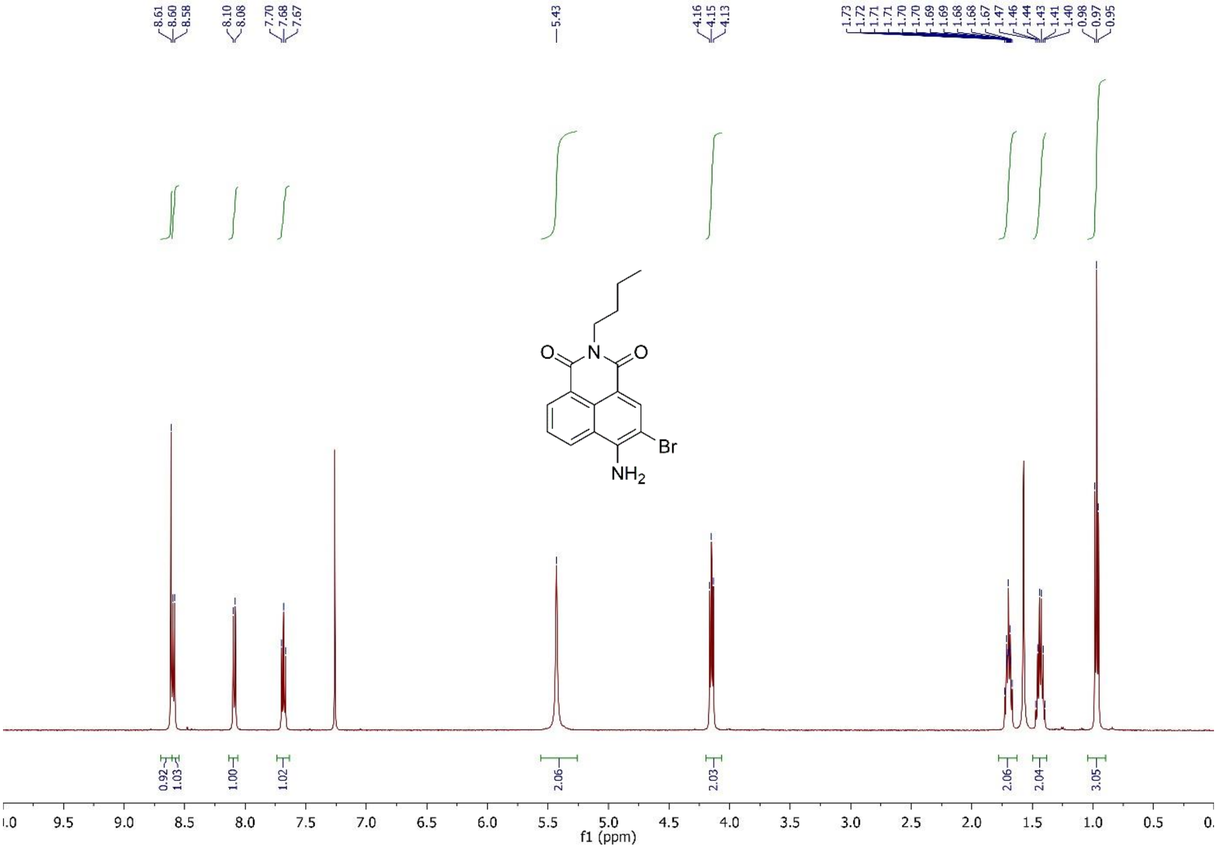

^{13}C NMR of compound 3

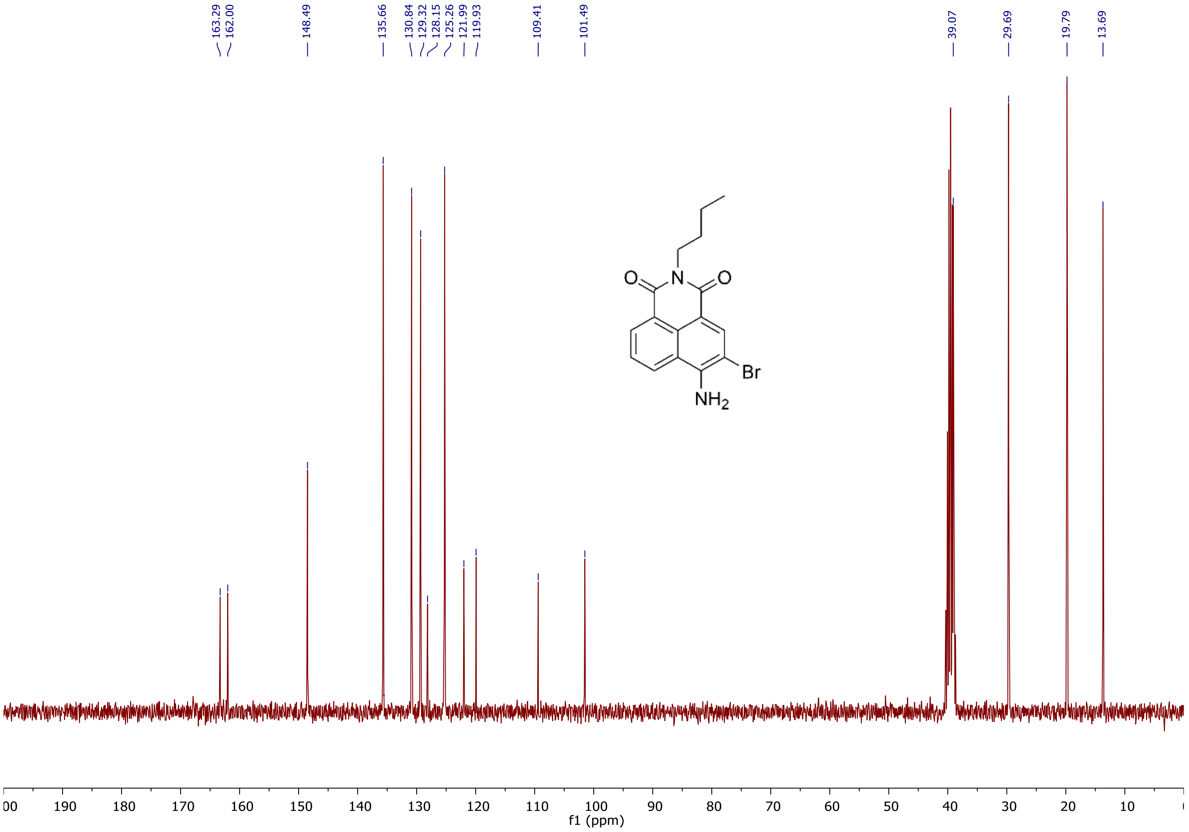

HRMS (ESI+) of compound 3

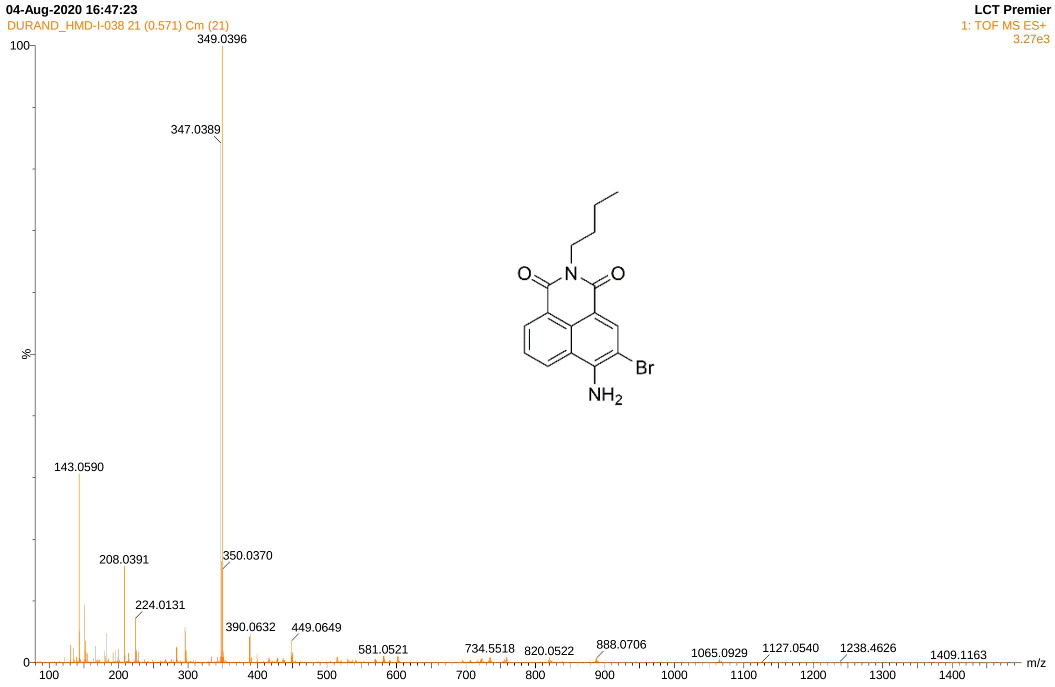

^{1}H NMR of compound 4

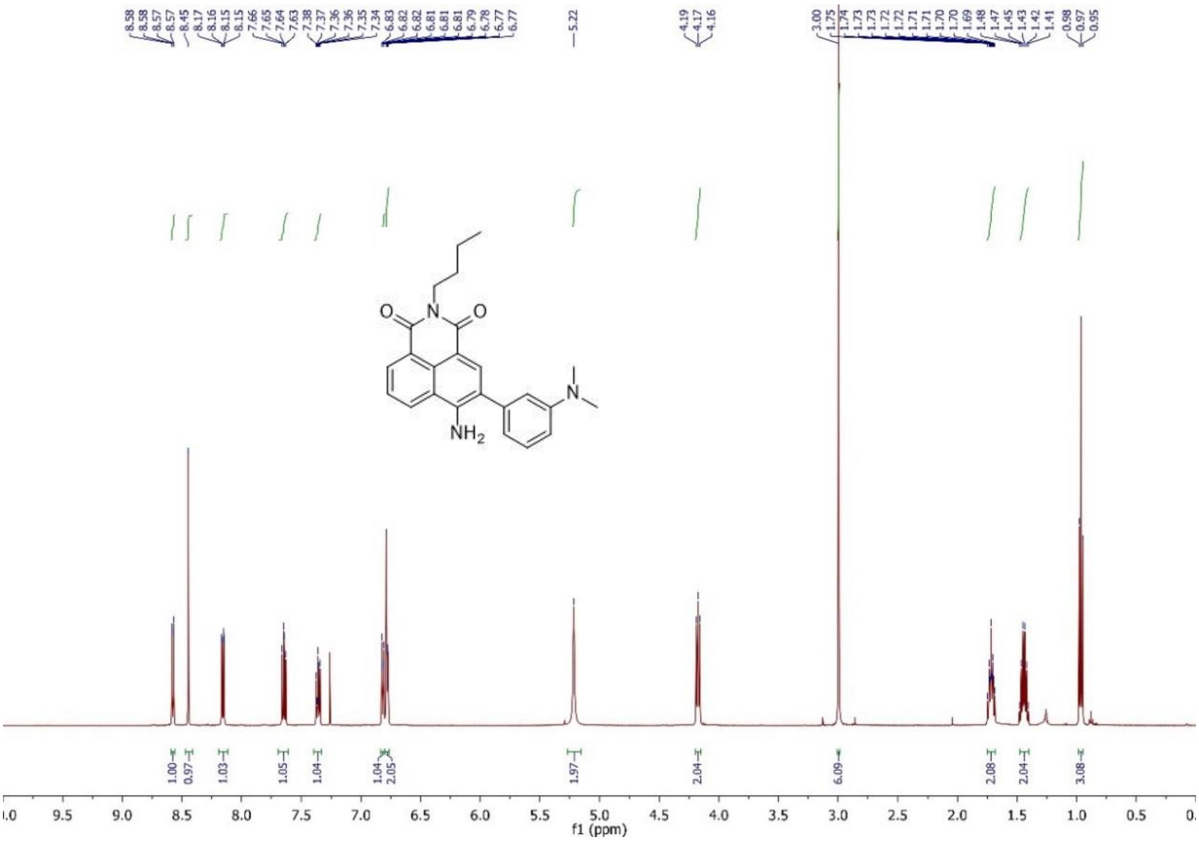

^{13}C NMR of compound 4

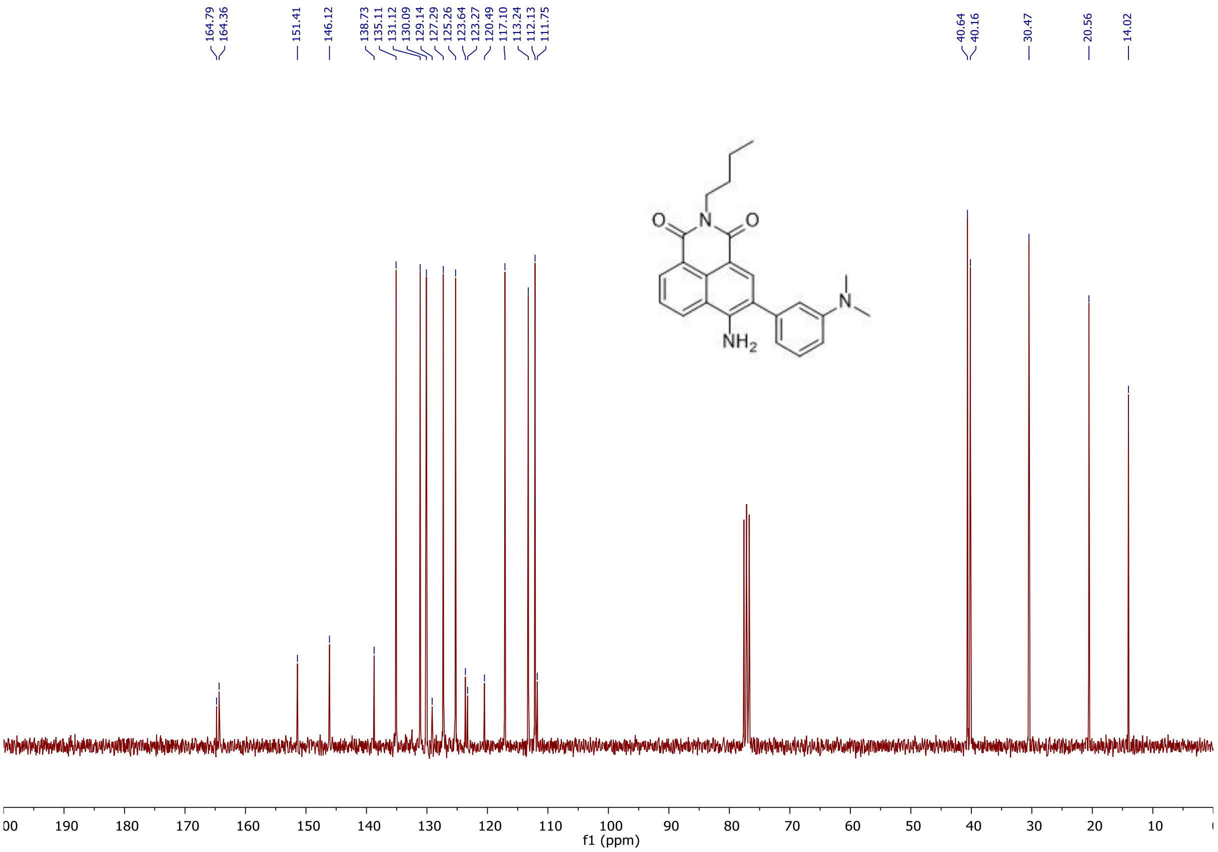

HRMS (ESI+) of compound 4

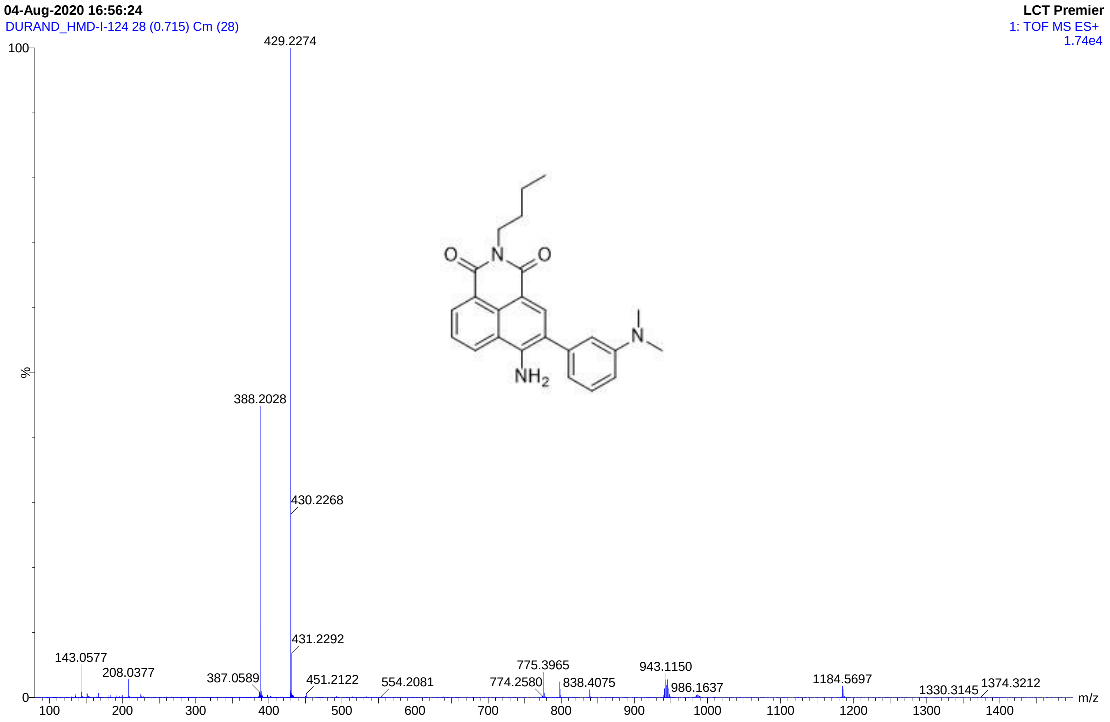

^{1}H NMR of compound 5a

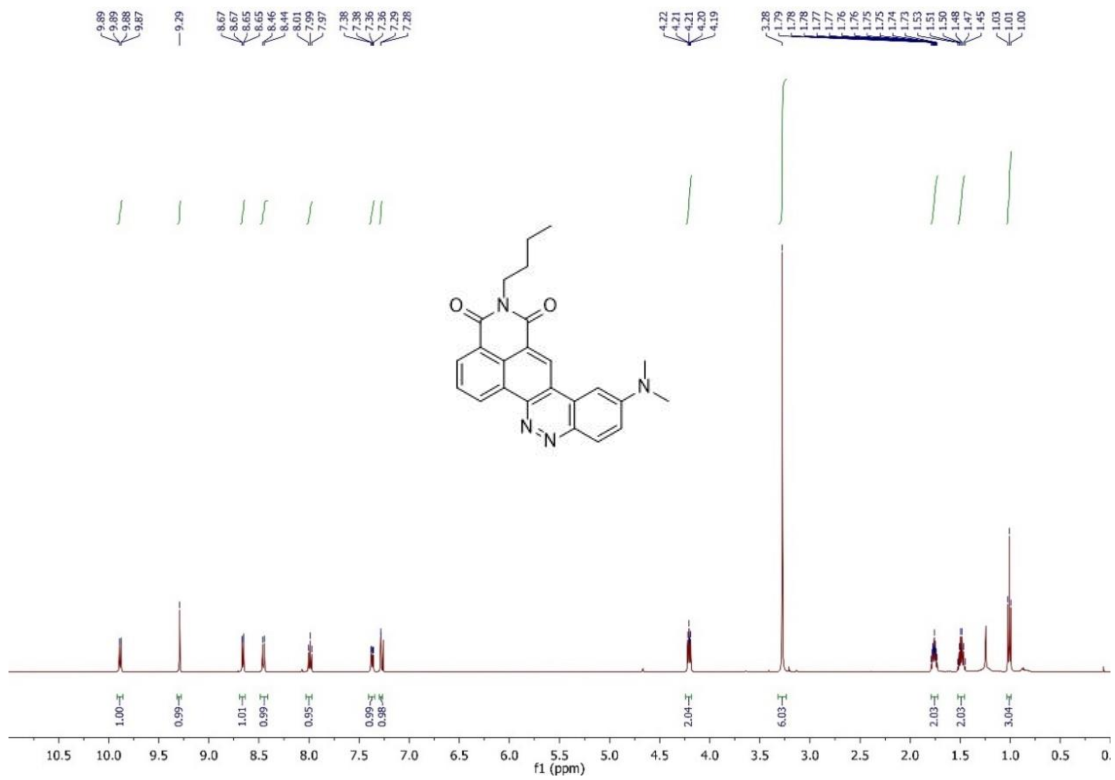

^{13}C NMR of compound 5a

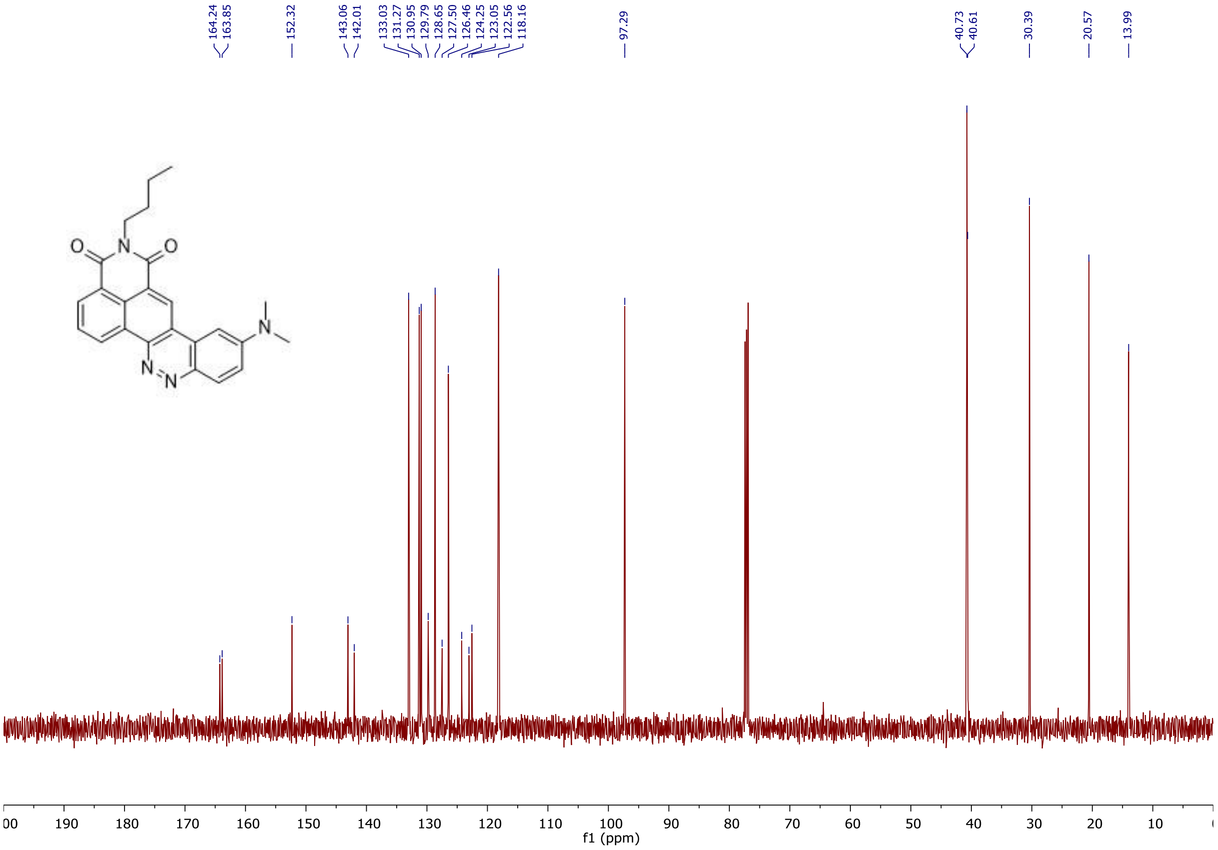

HRMS (ESI+) of compound 5a

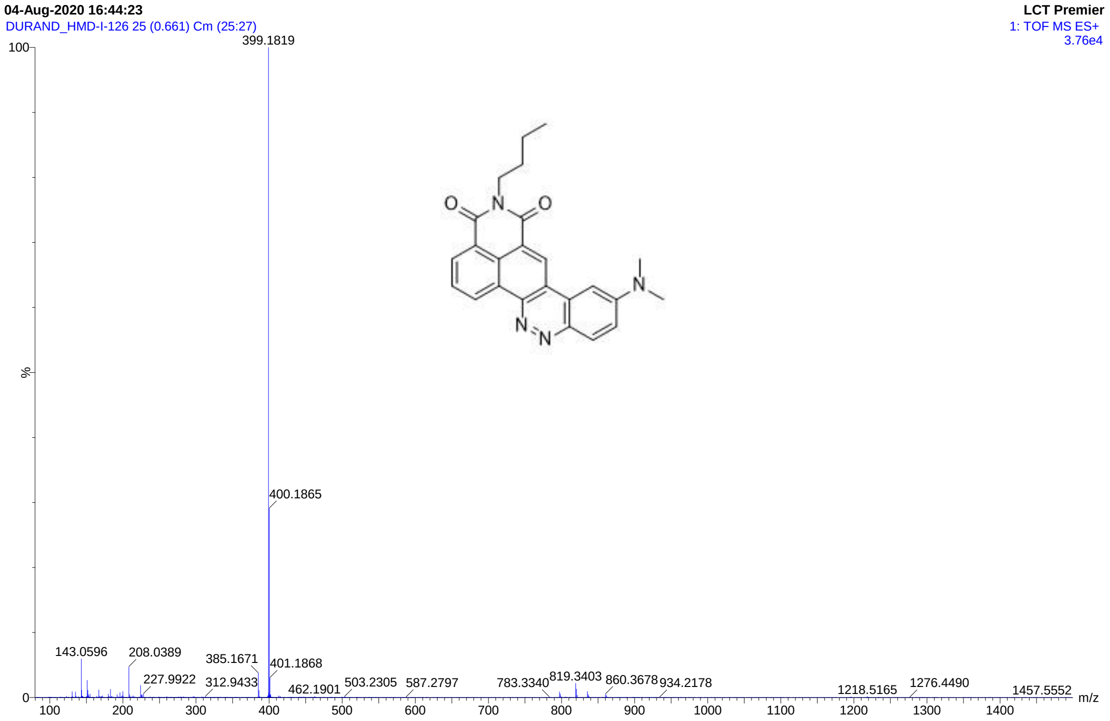

^{1}H NMR of compound 5b

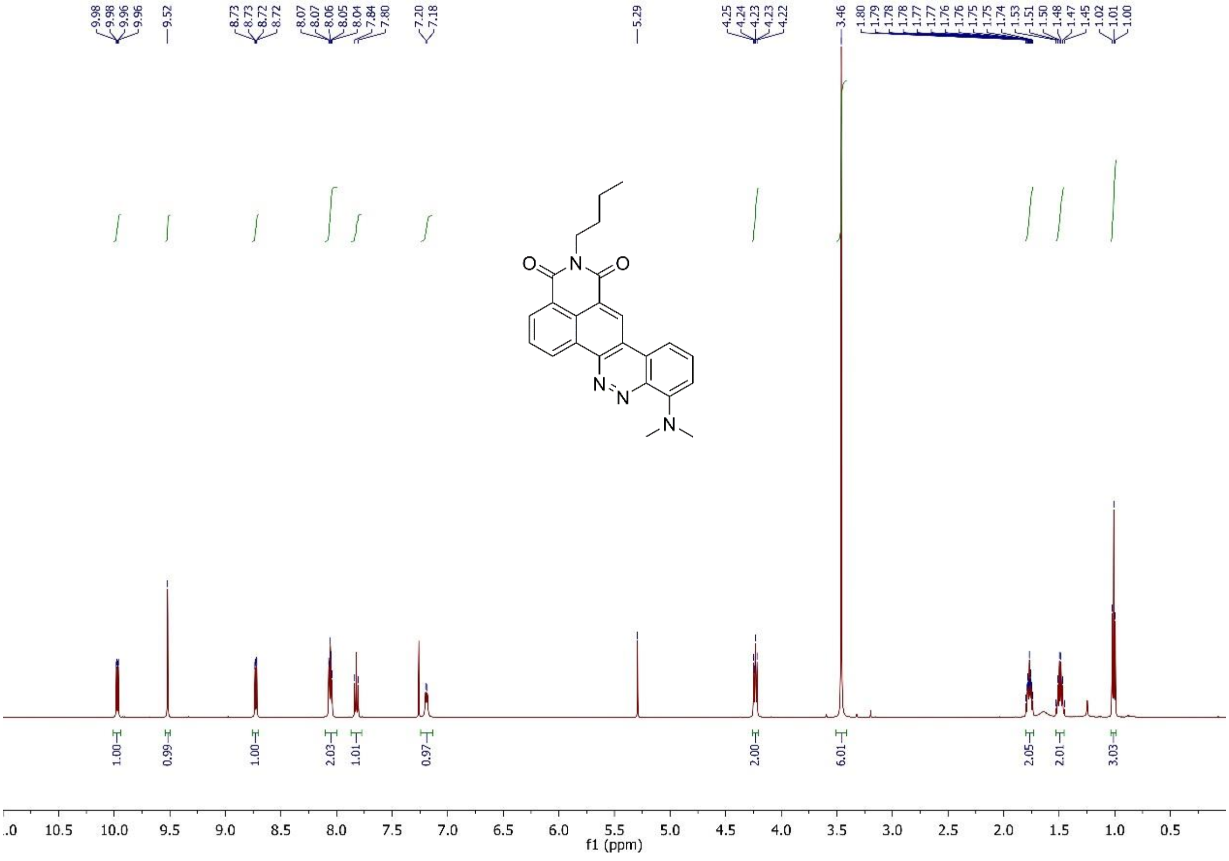

^{13}C NMR of compound 5b

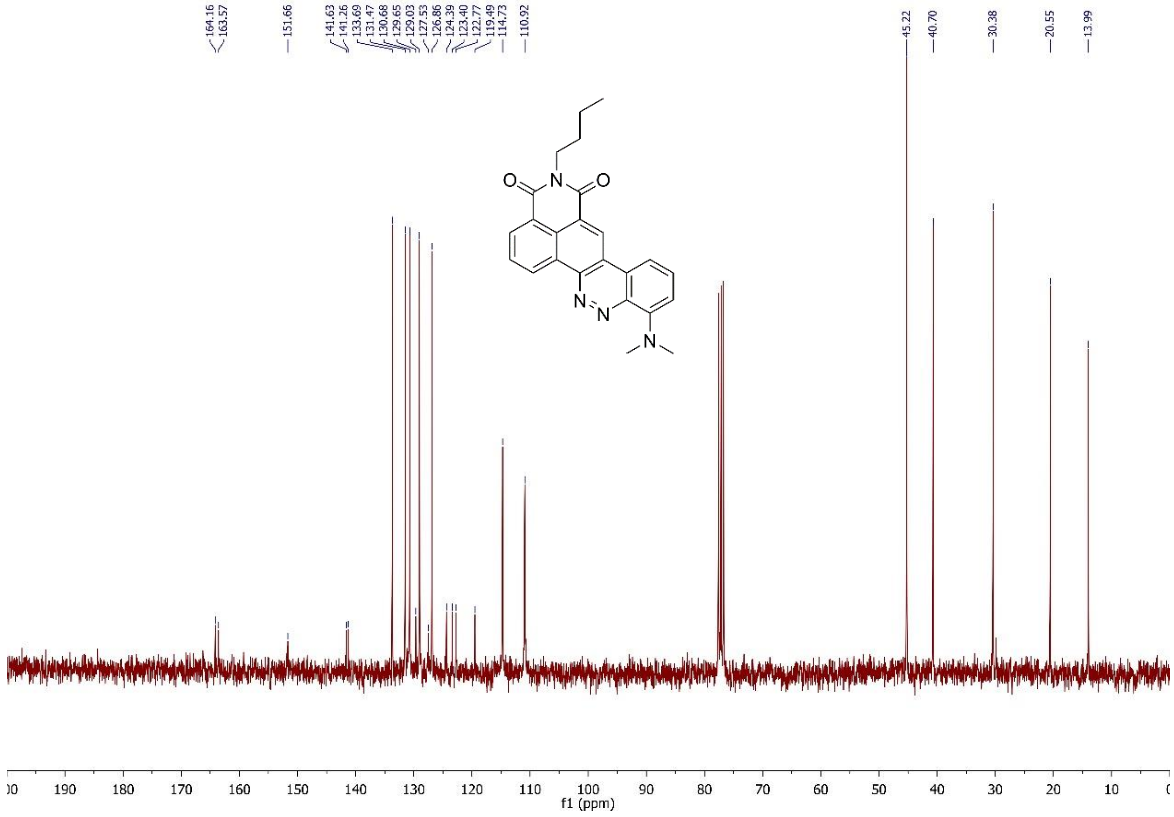

HRMS (ESI+) of compound 5b

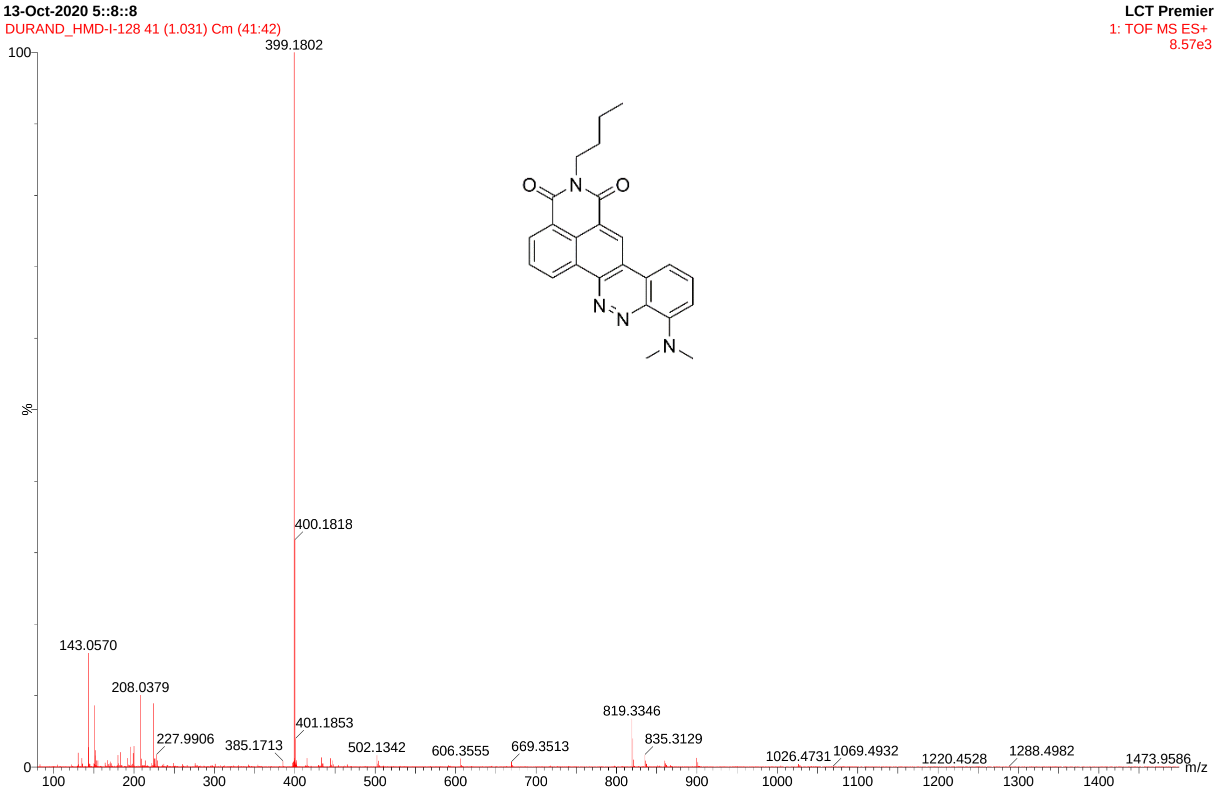

Optimized geometry of 5a

Label	Atom	X	Y	Z
1	C	2.916659	3.275333	-0.058929
2	C	2.592965	1.932524	-0.205713
3	C	1.241791	1.517535	-0.139009
4	C	0.214832	2.475402	0.078455
5	C	0.572515	3.835832	0.223486
6	C	1.901684	4.224527	0.155154
7	C	0.906655	0.137029	-0.287452
8	C	-1.157725	2.021989	0.142336
9	C	-1.460107	0.650558	-0.009233
10	C	-0.40212	-0.276417	-0.22317
11	C	-2.850525	0.278831	0.065949
12	C	-3.756252	1.35487	0.287742
13	H	3.959602	3.570801	-0.112857
14	H	-0.215738	4.561697	0.388685
15	H	2.163614	5.272862	0.268218
16	H	-0.603028	-1.335396	-0.340852
17	C	3.672798	0.940882	-0.430855
18	O	4.856234	1.256348	-0.483907
19	C	1.972604	-0.874352	-0.512248
20	O	1.725761	-2.071006	-0.631026
21	N	3.289304	-0.403904	-0.588148
22	C	4.358979	-1.396289	-0.819626
23	H	3.92786	-2.186854	-1.436352
24	H	5.145779	-0.887012	-1.378684
25	C	4.918536	-1.977579	0.484454
26	H	5.31008	-1.158753	1.102082
27	H	4.101949	-2.450989	1.045303
28	C	6.029267	-3.004255	0.224707
29	H	5.635893	-3.814108	-0.406675
30	H	6.837062	-2.528344	-0.349732
31	C	6.604625	-3.59871	1.515802
32	H	7.395038	-4.327839	1.300929
33	H	7.036157	-2.816849	2.153653
34	H	5.827079	-4.110712	2.097034
35	N	-2.115474	2.987138	0.354
36	N	-3.367061	2.660158	0.423525
37	C	-3.345436	-1.030457	-0.062048
38	C	-5.144218	1.078974	0.375684
39	C	-5.61819	-0.202285	0.249723
40	H	-6.684742	-0.377172	0.322171
41	C	-4.722737	-1.302545	0.024995
42	H	-2.6487	-1.840216	-0.230889
43	H	-5.81818	1.913566	0.545459
44	N	-5.211767	-2.58168	-0.09977

45	C	-4.295462	-3.690172	-0.328873
46	H	-3.574669	-3.794891	0.493875
47	H	-4.864706	-4.617899	-0.399646
48	H	-3.733564	-3.563014	-1.264615
49	C	-6.642501	-2.844612	-0.008905
50	H	-6.814784	-3.914139	-0.13602
51	H	-7.049955	-2.551595	0.96825
52	H	-7.204844	-2.317554	-0.791669

V. References

1. L. F. Yousif, K. M. Stewart and S. O. Kelley, *ChemBioChem*, 2009, **10**, 1939-1950.
2. M. J. Frisch, G. W. Trucks, H. B. Schlegel, G. E. Scuseria, M. A. Robb, J. R. Cheeseman, G. Scalmani, V. Barone, G. A. Petersson, H. Nakatsuji, X. Li, M. Caricato, A. V. Marenich, J. Bloino, B. G. Janesko, R. Gomperts, B. Mennucci, H. P. Hratchian, J. V. Ortiz, A. F. Izmaylov, J. L. Sonnenberg, Williams, F. Ding, F. Lipparini, F. Egidi, J. Goings, B. Peng, A. Petrone, T. Henderson, D. Ranasinghe, V. G. Zakrzewski, J. Gao, N. Rega, G. Zheng, W. Liang, M. Hada, M. Ehara, K. Toyota, R. Fukuda, J. Hasegawa, M. Ishida, T. Nakajima, Y. Honda, O. Kitao, H. Nakai, T. Vreven, K. Throssell, J. A. Montgomery Jr., J. E. Peralta, F. Ogliaro, M. J. Bearpark, J. J. Heyd, E. N. Brothers, K. N. Kudin, V. N. Staroverov, T. A. Keith, R. Kobayashi, J. Normand, K. Raghavachari, A. P. Rendell, J. C. Burant, S. S. Iyengar, J. Tomasi, M. Cossi, J. M. Millam, M. Klene, C. Adamo, R. Cammi, J. W. Ochterski, R. L. Martin, K. Morokuma, O. Farkas, J. B. Foresman and D. J. Fox, *Journal*, 2016.
3. G. R. Fulmer, A. J. M. Miller, N. H. Sherden, H. E. Gottlieb, A. Nudelman, B. M. Stoltz, J. E. Bercaw and K. I. Goldberg, *Organometallics*, 2010, **29**, 2176-2179.
4. K. Suzuki, A. Kobayashi, S. Kaneko, K. Takehira, T. Yoshihara, H. Ishida, Y. Shiina, S. Oishi and S. Tobita, *Phys. Chem. Chem. Phys.*, 2009, **11**, 9850-9860.
5. R.-J. Tang, T. Milcent and B. Crousse, *J. Org. Chem.*, 2018, **83**, 930-938.
